# Accurate Tracking of Locomotory Kinematics in Mice Moving Freely in Three-Dimensional Environments

**DOI:** 10.1523/ENEURO.0045-25.2025

**Published:** 2025-06-19

**Authors:** Bogna M. Ignatowska-Jankowska, Lakshmipriya I. Swaminathan, Tara H. Turkki, Dmitriy Sakharuk, Aysen Gurkan Ozer, Alexander Kuck, Marylka Yoe Uusisaari

**Affiliations:** Okinawa Institute of Science and Technology, Okinawa 904-0495, Japan

**Keywords:** CP55,940, harmaline, limb kinematics, motion capture, mouse, tremor

## Abstract

Marker-based motion capture (MBMC) is a powerful tool for precise, high-speed, three-dimensional tracking of animal movements, enabling detailed study of behaviors ranging from subtle limb trajectories to broad spatial exploration. Despite its proven utility in larger animals, MBMC has remained underutilized in mice due to the difficulty of robust marker attachment during unrestricted behavior. In response to this challenge, markerless tracking methods, facilitated by machine learning, have become the standard in small animal studies due to their simpler experimental setup. However, trajectories obtained with markerless approaches at best approximate ground-truth kinematics, with accuracy strongly dependent on video resolution, training dataset quality, and computational resources for data processing. Here, we overcome the primary limitation of MBMC in mice by implanting minimally invasive markers that remain securely attached over weeks of recordings. This technique produces high-resolution, artifact-free trajectories, eliminating the need for extensive post-processing. We demonstrate the advantages of MBMC by resolving subtle drug-induced kinematic changes that become apparent only within specific behavioral contexts, necessitating precise three-dimensional tracking beyond simple flat-surface locomotion. Furthermore, MBMC uniquely captures the detailed spatiotemporal dynamics of harmaline-induced tremors, revealing previously inaccessible correlations between body parts and thus significantly improving the translational value of preclinical tremor models. While markerless tracking remains optimal for many behavioral neuroscience studies in which general posture estimation suffices, MBMC removes barriers to investigations demanding greater precision, reliability, and low-noise trajectories. This capability significantly broadens the scope for inquiry into the neuroscience of movement and related fields.

## Significance Statement

Studying fine-scale motor behaviors in mice demands data with precision and fidelity that markerless approaches often struggle to provide. While marker-based motion capture is the gold standard for high-resolution kinematic analysis, its use in freely moving mice has been limited by challenges in marker use. This work overcomes these barriers by introducing implantable markers with replaceable reflective heads, fundamentally transforming the feasibility of robust high-definition 3D tracking across a wide range of behaviors and experimental conditions. By enabling the detection of subtle phenomena, such as harmaline-induced tremors, with spatiotemporal detail unmatched by markerless tracking, this approach provides a powerful tool for advancing studies of motor control and sensorimotor integration in rodents.

## Introduction

Currently, high-precision kinematic tracking in rodents necessary for probing (dys)function of movement-related neural circuits is largely based on restricting animal movement (Becker and Person, 2019) or creating artificial environments (such as transparent floors or locomotion in narrow confines) that can distort behavior and limit the translational potential of results. As the emerging translational crisis has highlighted weaknesses in animal experimental designs (Garner, 2014; De Schutter, 2019; Kennedy, 2022; Marshall et al., 2022), their improvement is needed to reflect more accurately the complexity of behavior. For example, it is rare for wild mice to explore flat and smooth surfaces, which are often used in experimental behavioral settings. Instead, the natural behavior of most rodents involves locomoting in uneven terrain with complex demands for body kinematics: they balance, climb, jump, and swim. Assessment of such 3D kinematics in freely behaving rodents requires the use of high-precision 3D motion capture.

The superior resolving power of marker-based motion capture (MBMC) in general, as compared to markerless systems, stems from the massive improvement in signal-to-noise ratio (by filtering out components unrelated to tracked points) as well as the on-camera compression of high-resolution image data to compact 2D coordinates. These aspects eliminate many of the signal processing bottlenecks necessary for markerless tracking. Furthermore, dedicated infrared illumination of retroreflective markers eliminates noise related to changing ambient lighting conditions.

Despite its precision, robustness, and common use in humans and other large animals (Ceseracciu et al., 2014; Schmitz et al., 2015; Nakano et al., 2020; Moro et al., 2022; Tang et al., 2024), the use of marker-based 3D motion capture in small animals such as mice and rats has been limited by technical difficulties (Becker and Person, 2019; Becker et al., 2020; Nakano et al., 2020; Steinebach et al., 2020; Topley and Richards, 2020; von Ziegler et al., 2021; Marshall et al., 2022) and markerless animal tracking has become the main tool for assessing behavior (Pereira et al., 2020; see, e.g., DeepLabCut, Mathis et al., 2018; Nath et al., 2019; Lauer et al., 2022; SLEAP, Pereira et al., 2022; 3-Dimensional Aligned Neural Network for Computational Ethology (DANNCE), Dunn et al., 2021; MoSeq, Wiltschko et al., 2020; Weinreb et al., 2024; LightningPose, Biderman et al., 2024). These approaches have recently experienced significant advances in flexibility and ease of use, but achieving precise 3D trajectory tracking remains a challenge (Karashchuk et al., 2021; Ito et al., 2022; Marshall et al., 2022; Li et al., 2023; Weinreb et al., 2024). Even in human studies, markerless techniques usually show only limited agreement with marker-based methods even under optimal conditions and typically generate mean errors of 10% (Schmitz et al., 2015; Buckley et al., 2019; Nakano et al., 2020; Steinebach et al., 2020; Topley and Richards, 2020; Drazan et al., 2021; Kanko et al., 2021; Ito et al., 2022; Moro et al., 2022; Wade et al., 2022; Li et al., 2023; Philipp et al., 2023; Song et al., 2023; Tang et al., 2024). Although dedicated markerless systems based on deep learning can reach accuracy comparable to marker-based systems (Bae et al., 2024), their performance depends on training data and variation between subject morphology (e.g., during aging), experimental conditions (e.g., subtle changes in lightning) and task characteristics (such as mode of locomotion) can lead to discrepancies. Importantly, the development of markerless technique accuracy is ultimately dependent on the comparison with ground-truth data, for which x-ray imaging (Moore et al., 2022) or MBMC data are needed.

In order to obtain precise kinematic recordings in mice that move freely in a three-dimensional environment, we took advantage of a marker-based 3D motion capture system (Qualisys; Dagenais et al., 2021; Roepstorff et al., 2021; O’Neill et al., 2022) conventionally used to track movement in humans and other large animals. Using a novel approach for permanent retroreflective skin-marker implantation as well as omitting enclosures between cameras and subjects that lead to unwanted reflections and spurious tracking errors, we obtain robust low-noise kinematic trajectories during various locomotory tasks without cumbersome machine learning-based post-processing commonly used to clean markerless or noisy marker-based trajectories (Marshall et al., 2021). Although the process of MBMC recording is somewhat more laborious than conventional behavioral video recording—requiring careful initial habituation of animals, consistent handling routines, and familiarization of experimenters with working on freely moving animals in the absence of enclosures—the quality of data and its ease of use (e.g., due to small raw file size) are transformative for the straightforward application of analytical approaches.

In this paper, we first quantify the tracking performance of MBMC in terms of continuity (gaps), accuracy, and noise (“jitter”) using mice fitted with skin-implanted, retroreflective markers during exploration of a relatively conventional open field (OF) recording arena. Next, we demonstrate how the MBMC approach facilitates examination of mouse movements across various spatiotemporal scales, such as simultaneous assessment of general locomotion and fast limb movements. Our findings reveal previously unrecognized variations in mouse limb dynamics during different locomotor scenarios. We also show that even well-known drug effects (such as those observed after cannabinoid receptor activation; Patel and Hillard, 2001) can be surprisingly different when examined in horizontal versus vertical locomotion, underscoring the importance of employing a broader spectrum of behavioral settings. Finally, to showcase the resolution of MBMC, we track harmaline-induced tremor and show a significant correlation of motion across the whole animal body.

## Methods

### Experimental subjects and drug administration

Adult male C57BL/6A mice (CLEA Japan), aged 10–12 weeks (20–25 g) at the beginning of the experiments, were used. Mice (*n* = 29) were subjected to systemic intraperitoneal drug administration followed by behavioral assessments. The animals were randomly assigned to drug treatments and tested in a counterbalanced Latin square within-subject design, with at least a 72 h washout period between treatments.

Drug treatments were selected to induce locomotor disturbances through different mechanisms of action. The CB_1_ and CB_2_ receptor agonist CP55,940 (Tocris), known to inhibit locomotor activity at doses of 1 mg/kg, was administered at a low dose of 0.3 mg/kg to test whether subtle changes in fine kinematics could be observed and to evaluate the sensitivity of the motion capture system. Harmaline, known to induce rhythmic activity in the inferior olive and produce whole-body tremor, was administered at a high dose of 20 mg/kg to examine the tremor-tracking potential of MBMC. Doses were selected based on preliminary experiments and existing literature (Martin et al., 2005; Ignatowska-Jankowska et al., 2015a, 2015b).

The drugs were dissolved in a vehicle solution consisting of ethanol, Kolliphor, and saline (1:1:18 ratio) and administered at volumes of 10 μl/g for CP55,940 and 20 μl/g for harmaline. All drugs were administered 30 min before recordings. Investigators were blinded to experimental conditions whenever possible.

The animals were housed in a temperature (20–22°C) and humidity (55 ± 10%) controlled, Association for Assessment and Accreditation of Laboratory Animal Care (AAALAC)-approved facility, with a 12/12 h reversed light/dark cycle (lights on at 0700 h and off at 1,900 h). They were kept in enriched environments with *ad libitum* access to food and water. All experiments were conducted during the dark (active) phase of the circadian cycle. This study is reported in accordance with Animal Research: Reporting of In Vivo Experiments (ARRIVE) guidelines (Du Sert et al., 2020).

### Marker implantation procedure

Marker implantation was performed 11–14 d before the start of behavioral experiments to ensure full healing. We used male C57BL/6A mice (CLEA Japan) aged 10–12 weeks and weighing 20–25 g at the time of implantation. Distances for marker placement should be adjusted when working with larger or smaller animals.

Markers were placed across the shoulder blades, lumbar spine, hips, and along the tibia between the knees and ankles. These locations were selected to enable tracking of whole-body movement during locomotion, postural adjustments, and finer kinematic features such as tremor, miscoordination, or swaying.

The total weight of all uncoated markers (1,260–1,270 mg) remained within 5–7% of the animals’ body weight. The implants caused no visible discomfort, remained stable for over a year, and enabled repeated within-subject testing. Their subcutaneous anchoring to connective tissue ensured more faithful tracking of skeletal movement than skin-surface markers, which are susceptible to slippage on the loose rodent skin.

Under isoflurane anesthesia (2–3%, Somnosuite, Kent Scientific), the fur around planned implant sites was shaved. Mice were positioned symmetrically on the surgical platform with hind legs bent such that the feet formed a line perpendicular to the body’s long axis. The skin was cleaned with 70% ethanol, and anatomical landmarks were marked—specifically, the midline between the hips and between the shoulder blades.

**Figure 1.**
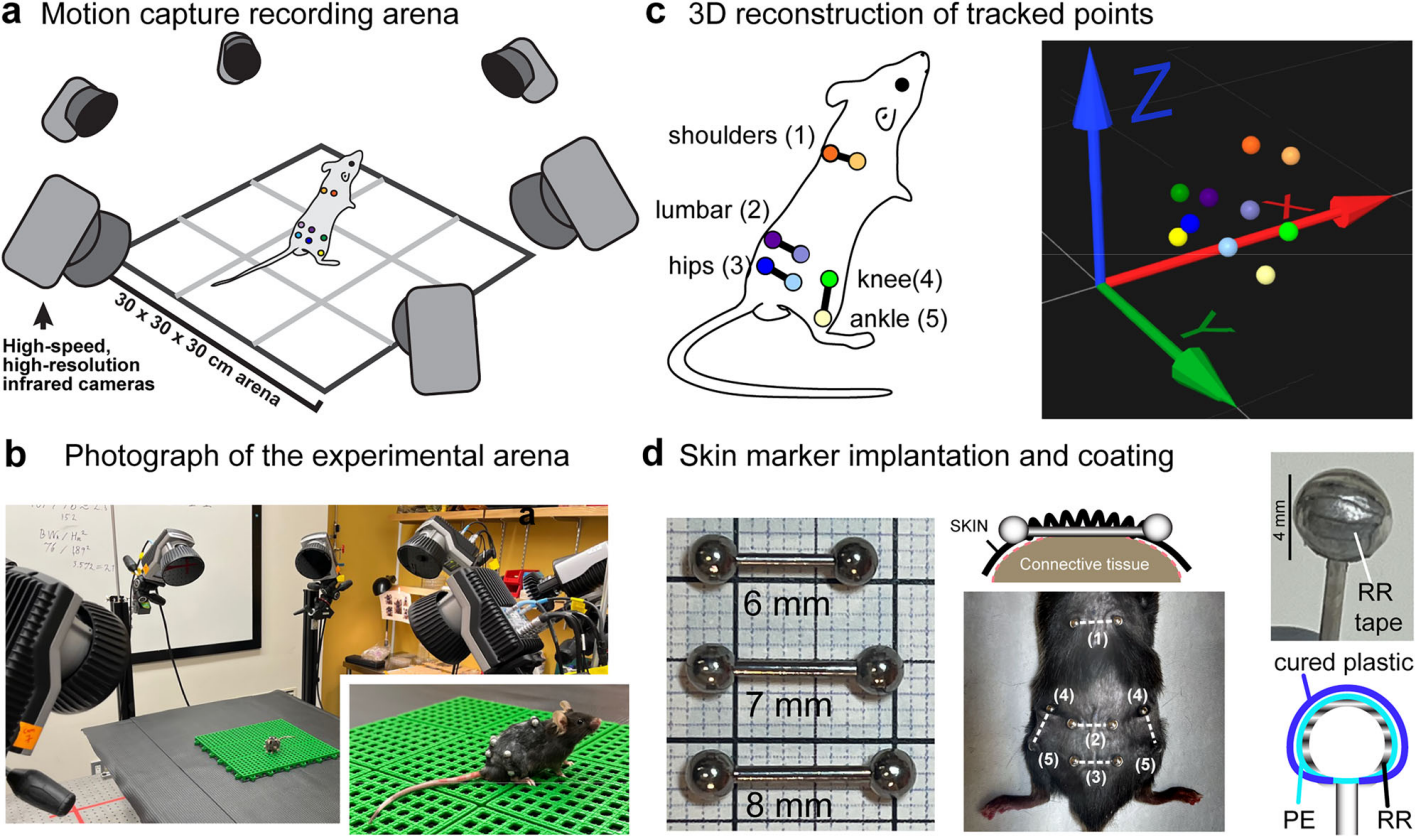
Realization of MBMC in mice using barbell-shaped underskin marker implants. ***a***, Schematic of the motion capture recording set up; for actual scale see photograph in ***b***. Inset shows a close-up of a mouse wearing retroreflective markers during a recording session. Note the relaxed posture of the animal despite the absence of enclosure. ***c***, Schematic and labeling of marker positions used in this study (left) and a 3D reconstruction of marker positions from the QTM software. ***d***, Construction and attachment of skin-marker implants. Three different lengths of barbell piercings were used as most fitting for a given body part (left). The shaft of the barbell piercing is inserted through and under the skin (middle). During motion capture recordings, the stainless steel 3 mm spheres of the piercings are replaced with 4 mm spheres that have been covered with retroreflective (RR) tape, polyethylene (PE) plastic film, and coated with UV-cured plastic (right).

A total of five pairs of stainless steel markers were implanted subcutaneously at designated body locations (Fig. 1). Each implant consisted of a short steel rod (<14 mm in length, 0.9–1.27 mm in diameter; 18–20 gauge), capped with 3–4 mm diameter screw-on stainless steel spheres. We used 6 mm piercings for the shoulder and lumbar implants, 7 mm for the hips, and 8 mm for the legs.

A calibrated stick (e.g., a Q-tip marked using precise calipers) was used to mark the placement of the paired holes. To ensure implant stability and reduce rejection, the skin bridge between holes was made to be at least twice the length of the piercing shaft. For example, for hip markers spaced 14 mm apart, symmetrical dots were drawn centered on the midpoint of the hips. For lumbar spine placement, a dot was marked 8–10 mm above the hip center, and paired lumbar holes were placed 14 mm apart.

For the upper leg markers, the knee position was marked with the leg in both maximally bent and half-bent positions. The hole was placed between these two knee positions to accommodate joint movement. The lower leg marker was positioned 14 mm above the heel, centered on the calf. The total distance from heel to upper leg marker was approximately 28 mm. Symmetry of all markings was verified before piercing.

Careful placement of the lower leg holes is essential: holes placed too laterally may allow the marker to slide behind the leg, making it invisible to cameras. The lower leg marker must be centered on the calf, and the ankle marker positioned high enough to avoid ground contact when the mouse stands, preventing interference with walking.

At each marked location, a small (<1.5 mm) skin puncture was made using an 18G sterile needle. Each hole was then widened with super-fine forceps (e.g., Dumont #55), and the disinfected steel rod part of the piercing (soaked in 70% ethanol for at least 20 min) was inserted. The piercing was then secured with a screw-on ball on the opposite end.

All implants were cleaned with ethanol and dried before insertion. During implantation, the skin was gently lifted using forceps to minimize trauma. An excess fold of skin was left between the balls of each piercing, which naturally adjusted during healing.

Mice were allowed to recover for 11–14 d before recording sessions began. During this period, behavioral training was initiated, but implant exchange or manipulation was avoided to ensure proper healing.

### Behavioral training

To ensure accurate 3D tracking, no transparent surfaces or obstructions (e.g., transparent walls) can be present between the cameras and the experimental subject, as spurious reflections and distortions catastrophically degrade tracking performance (Fig. 1*a,b*). Consequently, animals must be habituated to the naturally anxiogenic OF arena and so they will remain within the designated area during testing without restriction of movement. We developed a dedicated protocol for animal training to achieve these goals.

#### Handling and pre-training

Experiments began 11–14 d after marker implantation to allow proper wound healing and stable marker positioning. During this period, mice undergo handling, habituation, and task training in the following sequence:

1. Handling sessions, lasting 2–3 d, familiarized mice with human interaction and included several steps. The animals were first taken out of their cages using a tube and then gently pulled by the base of the tail to the outer part of the experimenter’s hand or forearm. This progressed to scooping the mice while holding their tails gently and finally to scooping without tail support.
2. Habituation in the arena for 5–7 d: Initially, mice were placed in a circular 30 cm arena with 4 cm walls in a brightly lit room for 2–3 d. In the next phase lasting 3–4 d, mice were placed in a circular arena without walls. If they attempted to leave the arena, they were guided back by gently pulling their tails or, if only partially leaving, by tapping their noses. The training lasted 2–5 min per session. The training arena was distinct from the experimental arena to prevent overhabituation and ensure robust exploratory behavior during experiments.
3. Task training followed habituation and included two components: vertical climbing on a wheel (CLB) and running on a treadmill (TRM).

Each animal was subjected to the level of training that resulted in a similar performance, and all animals are trained until they correctly perform the task as expected. Positive or negative reinforcements were not used to facilitate training.

#### Marker exchange process

During the training period, the mice were also accustomed to the 3- to 5-min process of manual marker exchange to avoid the need for anesthesia. Outside recording periods, the mice wore uncoated 3 mm steel screw-on spheres. These were exchanged for larger retroreflective markers 15 min before recording and replaced with the original spheres after the session.

#### Behavioral experiment apparatuses

Behavioral assessments were conducted within a 30 × 30 × 30 cm volume surrounded by cameras. The experimental apparatuses were placed within this volume. For OF task, a 30 × 30 cm textured polyethylene surface was used as the “arena”. For the CLB task, the outer surface of a spoked running wheel (25 cm in diameter) designed for rats was used. The wheel was manually moved to match voluntary mouse movements (Movie 3), and retroreflective markers were placed on its outer rim to track movement. TRM running tasks were conducted on a motorized single-lane treadmill (MazeEngineers), with markers attached to the belt at 20 cm intervals to monitor speed (Movie 5). All apparatuses were cleaned between trials.

### Retroreflective marker fabrication

To achieve high retroreflectivity and durability suitable for rodent experiments, 4 mm stainless steel barbell piercing screw-on balls (Fig. 1*d*; Felio Co., Ltd.) were used as the base. These markers were coated with three layers: (1) retroreflective tape (3M), (2) linear low density polyethylene film (TRUSCO Micron 25 X W X/300 m, TSF2550), and (3) UV-curable plastic (BD-SKCJ).

Retroreflective tape was cut into narrow strips (6 mm width) and shaped into slightly curved crescents approximately 1 mm wide. The strips were applied to the marker with the edges slightly overlapping, covering the surface from back to front in the coronal plane. A round area of uncovered metal on top of the sphere, approximately 1–2 mm in diameter, was further covered with a circular piece of reflective tape (2–4 mm in diameter) to ensure uniform reflectivity.

To protect the fragile retroreflective tape from damage, a UV-curable plastic layer was used as the outermost coating. However, direct contact with liquids or media other than air distorts the reflectivity of the tape, rendering it ineffective. To preserve a layer of air between the plastic and the retroreflective tape, the tape-covered spheres were first wrapped with polyethylene film. Finally, liquid UV-curable plastic was applied as the third and outermost layer and hardened using UV light. To maintain the sphericity of the markers, crucial for radial reflection, the markers were continuously rotated during the plastic curing process. Gloves were worn throughout the procedure to prevent fingerprints on the surface, which could degrade the reflectivity.

Alternative methods for coating the screw-on spheres, such as retroreflective paints and sprays, were explored, but they either did not provide a sufficiently strong retroreflective signal on the small surface of the sphere or lacked the durability required for rodent experiments. After extensive testing, the finalized marker design demonstrated strong signal detection by cameras, excellent durability, a damage-resistant surface, and lightweight properties.

### Marker quality testing

The quality of the retroreflective markers was tested before attempting to use them in experiments. Individual markers were placed on a custom well-plate made from black, non-reflective material in the motion capture arena, and tracking tests were conducted using well-calibrated cameras. Each marker was subjected to random shaking for 20 s while being tracked. This process was repeated three times, with the well-plate positioned differently for each trial.

During these “quality control recordings”, the exposure setting for the motion capture was reduced to half (25*u*s; frame rate kept at 300 fps) of the exposure used in experimental conditions. Markers were deemed acceptable for experimental use only if they were tracked without gaps throughout all three quality control trials. Markers that did not pass the tests were discarded.

### Behavioral tracking using MBMC

Motion capture recordings were conducted using the Qualisys Oqus 7+ camera system (Qualisys; Josefsson et al., 1996). Standard 22 mm lenses were replaced with 40 mm lenses, optimized for small tracking volumes (focus distance during recordings: 39.5 mm; aperture: 2.8). Retroreflective markers were stroboscopically illuminated with infrared LED ring lights attached to the cameras (exposure time: 50 μs; frame rate: 300 fps). Six cameras were positioned at 60° angles relative to each other, with a slight downward tilt to optimize the capture of all markers. For the CLB task, only four cameras were needed for tracking. Importantly, as the Qualisys motion capture cameras only produce marker coordinate data, it is necessary to complement the recordings with a well-placed conventional video camera to allow examination of the non-tracked body parts.

To ensure the high quality of the triangulated positional data, the cameras were regularly calibrated following standard procedures (https://docs.qualisys.com/getting-started/content/getting_started/running_your_qualisys_system/calibrating_your_system/calibrating_your_system.htm). Briefly, during calibration, the spatial arrangement and lens properties of each camera are mathematically characterized to accurately reconstruct marker positions from multiple camera images through triangulation algorithms (e.g., direct linear transformation methods; Hartley, 2003). Calibration was deemed successful when all cameras reported average residuals below 0.2 mm.

Although lighting and other environmental conditions do not directly affect marker tracking accuracy because of dedicated infrared illumination, they can influence animal behavior in open environments. Therefore, basic experimental precautions were taken, including keeping ambient lighting conditions stable, minimizing noise, and restricting personnel movements in the recording space. Importantly, the level of environmental control needed may vary depending on how thoroughly animals are habituated to the experimental conditions; in our experience, well-habituated animals exhibit stable and naturalistic behaviors even under moderate changes in experimental conditions.

At the beginning of each recording, mice were placed in the arena used in training period to confirm that all markers are clearly visible to at least three cameras using the experimental recording parameters (300 fps with 50 μs exposure time). After completion of an experimental day, the marker trajectories were manually labeled using default settings in Qualisys Track Manager (QTM) software (Qualisys; 2022 version) and exported to MATLAB for further analysis. Animations of 3D marker position reconstructions shown in the movies are generated in the QTM software and exported. The final movies were compiled in DaVinci Resolve 19 (Blackmagicdesign).

### Gap filling and glitch removal

Minimal post-processing was applied to marker trajectories only when necessary to ensure data continuity for computational analysis. Short gaps (shorter than 50 frames; ∼165 ms) were bridged using linear interpolation, producing continuous trajectories essential for calculations requiring uninterrupted data (e.g., windowed speed estimation). Longer gaps, which occurred rarely, were excluded from subsequent analyses to avoid introducing artifacts. Importantly, gap-filled sections were not used in analyses of kinematic features such as step heights.

To address occasional short “jitter”—sharp, transient deviations caused by positional reconstruction ambiguity—a simple algorithm was employed. Jitter events were defined as localized changes that exceeded 0.05 mm in peak prominence and lasted for no more than three frames (∼10 ms). The affected data points, along with their immediate neighbors, were replaced with interpolated values to restore the continuity of the trajectory. This process ensured that only clearly erroneous and short-lived artifacts were corrected, preserving the integrity of the overall trajectory.

### Motion capture performance quantification

The accuracy and reliability of the motion capture system were evaluated using three key metrics: marker visibility, residuals, and positional error. These measures collectively define the system’s accuracy and robustness in reconstructing 3D trajectories from markers on freely behaving animals.

**Figure 2.**
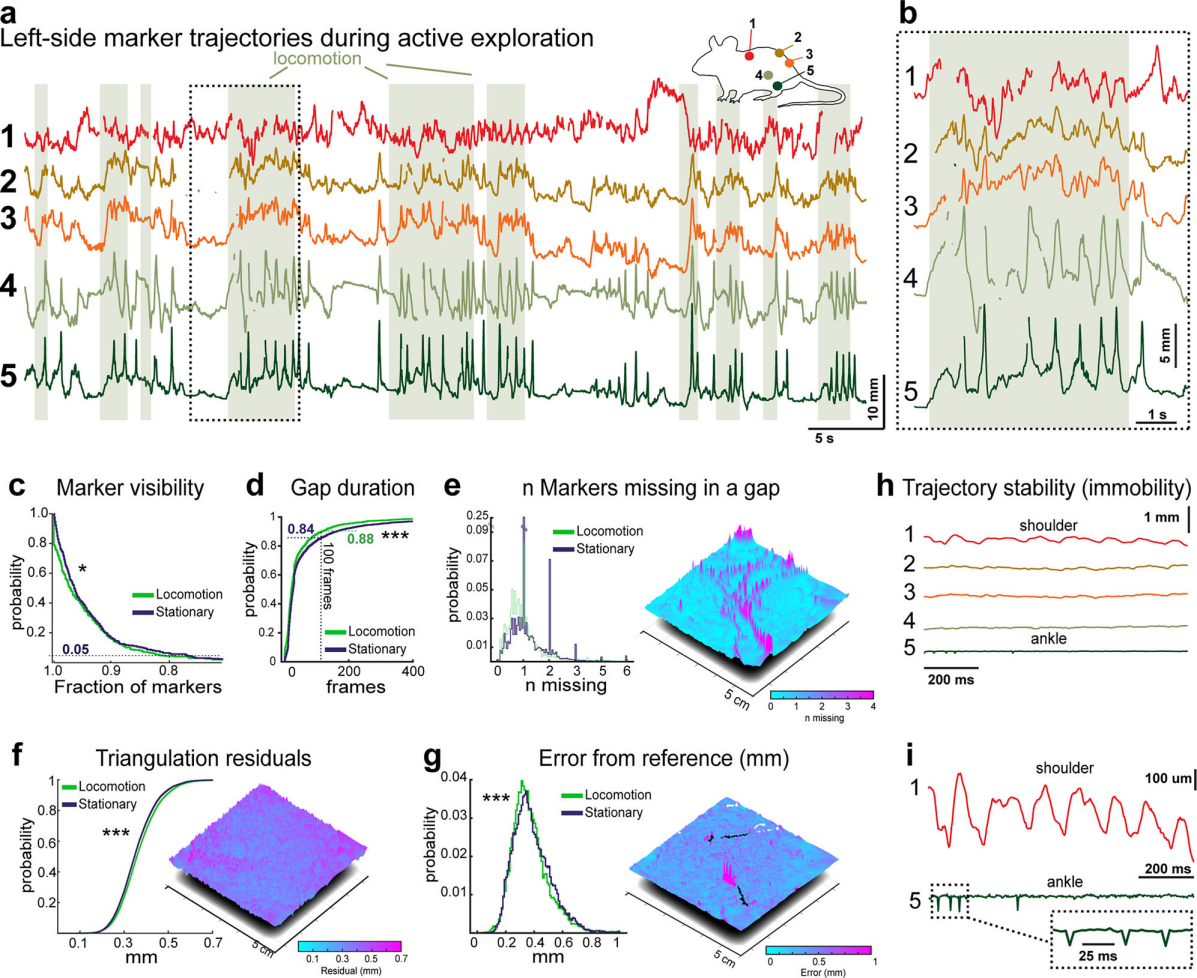
Characterization of the robustness, precision, and accuracy of motion capture tracking. ***a***, Representative example of raw vertical trajectories obtained from five markers on left side of the body. Schematic displays the color code for markers. Shaded green regions indicate periods when the mouse was locomoting. ***b***, Portion of trajectories indicated by dashed rectangle is shown in higher temporal resolution, displaying short duration of missing data. ***c***, Marker visibility shown as the probability that given fraction of markers is visible in a frame. The results are shown separately for locomotory and stationary periods. ***d***, Duration of tracking gaps shown as cumulative probability distributions for locomotory and stationary periods. Dashed lines highlight the probabilities that gap duration is less than 100 frames (∼300 ms). ***e***, Probability for locomotory and stationary periods (left) and spatial distribution on the square recording arena (right) of missing markers counts, smoothed over 100 frames (∼300 ms). The individual peaks in the right panel correspond to a single animal with somewhat worse marker quality. ***f***, Probability (left) and spatial distribution (right) of the triangulation residuals obtained from raw Qualisys tracking results. ***g***, Probability (left) and spatial distribution (right) of the tracking error, quantified as the difference from known reference distance between pairs of markers. ***h***, Representative vertical trajectories of all markers on one side of a mouse during a period of immobility. ***i***, Shoulder blade and ankle trajectories from (h) shown in higher spatial resolution. Note oscillations of 100–300 μm seen in shoulder marker possibly reflecting breathing movement. The dashed rectangle region from ankle marker is shown with expanded temporal resolution in the inset, showing unitary, 100 μm “glitches”. *p < 0.05; ***p < 0.0001, Mann–Whitney U test.

- Marker visibility (Fig. 2*c*–*e*) quantifies the percentage of frames in which each marker is successfully detected during a recording session. High visibility across frames indicates consistent detection and tracking of markers, ensuring reliable trajectory data.
- The residuals (Fig. 2*f*) are generated by Qualisys QTM software and represent the average differences between the 2D marker rays that contribute to the reconstruction of a single 3D point (Qualisys, 2024).
- Tracking error magnitude (Fig. 2*g*) was calculated for each pair of markers based on the absolute frame-by-frame differences between the distance measured between pairs of markers and the known distance between them. Assuming the two markers contribute equally to the error, the per-marker error was estimated as half of the pairwise error.

### Kinematic measurement explanations and definitions

**Figure 3.**
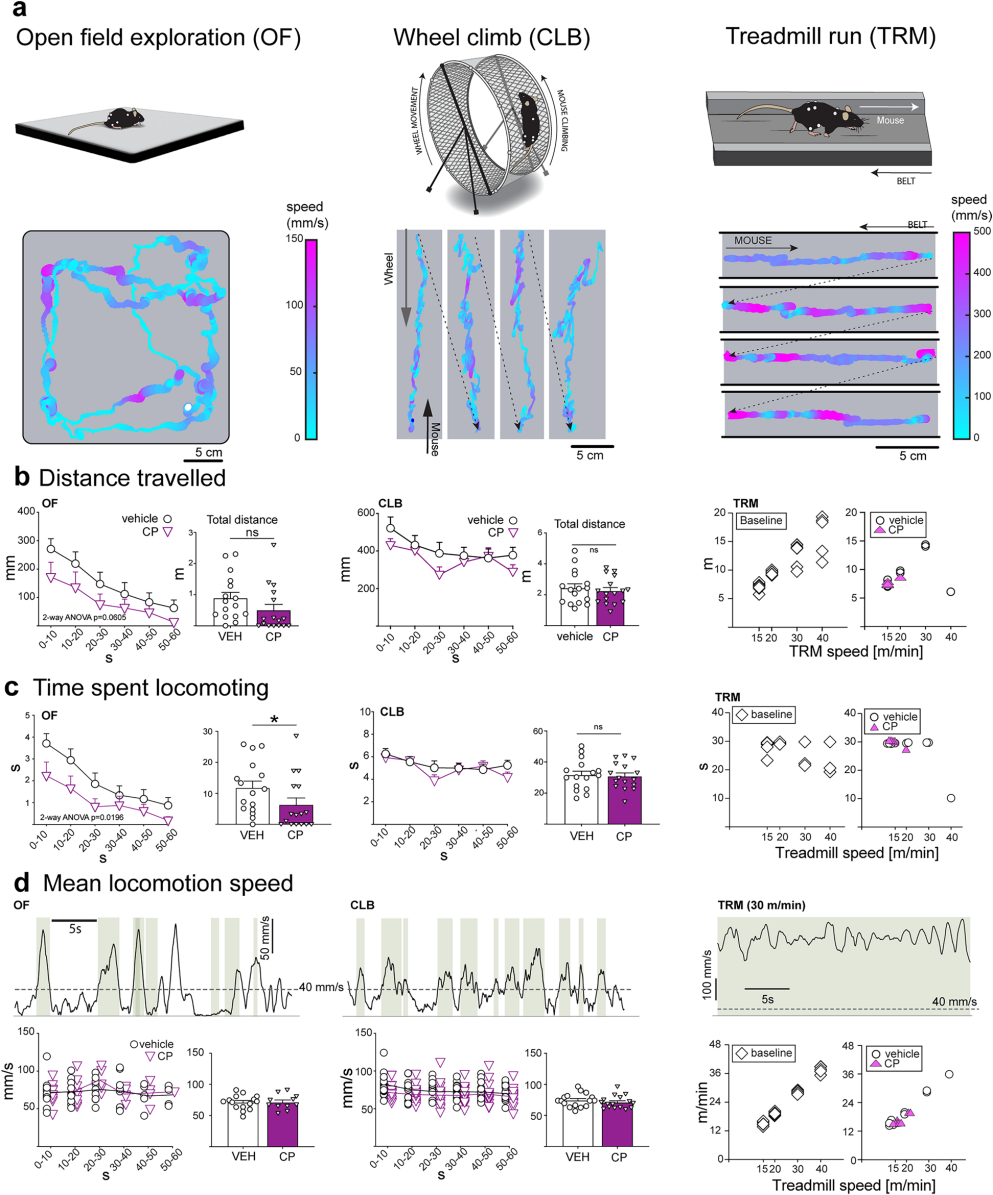
Using motion capture to monitor general activity parameters in 3D environments. ***a***, Schematic description of the three locomotory behaviors used in this work (top panels) and representative example trials (bottom panels). Animal position is shown as the midpoint of hip markers, color coded with speed. Position on wheel and treadmill is shown with respect to the moving substrate (wheel or treadmill band, respectively). ***b***, Effect of CP55,940 (CP) on distance traveled during full trials shown binned means of all animals + SEM (left panels) and as total values for full trials (right panels). For treadmill data, both baseline (no injection) and vehicle-injected (VEH) groups are shown. ***c***, Top panels: representative trial speed profiles from the three behaviors. Shaded green regions denote periods when mouse was locomoting (mean speed higher than 40 mm/s, indicated by dashed line). ***c***, Bottom panels: binned means for individual animal speeds while locomoting (left panels) and mean locomotory speeds over whole trials (right panels), shown for VEH and CP groups. For treadmill data, mean trial speeds shown for each animal, in baseline, VEH, and CP groups. *p < 0.05 in paired *t* test. ***d***, Time spent locomoting shown binned (left panels) and over whole trials (right panels). Note that the only metric reaching statistical significance is time spent locomoting in the OF.

**Figure 4.**
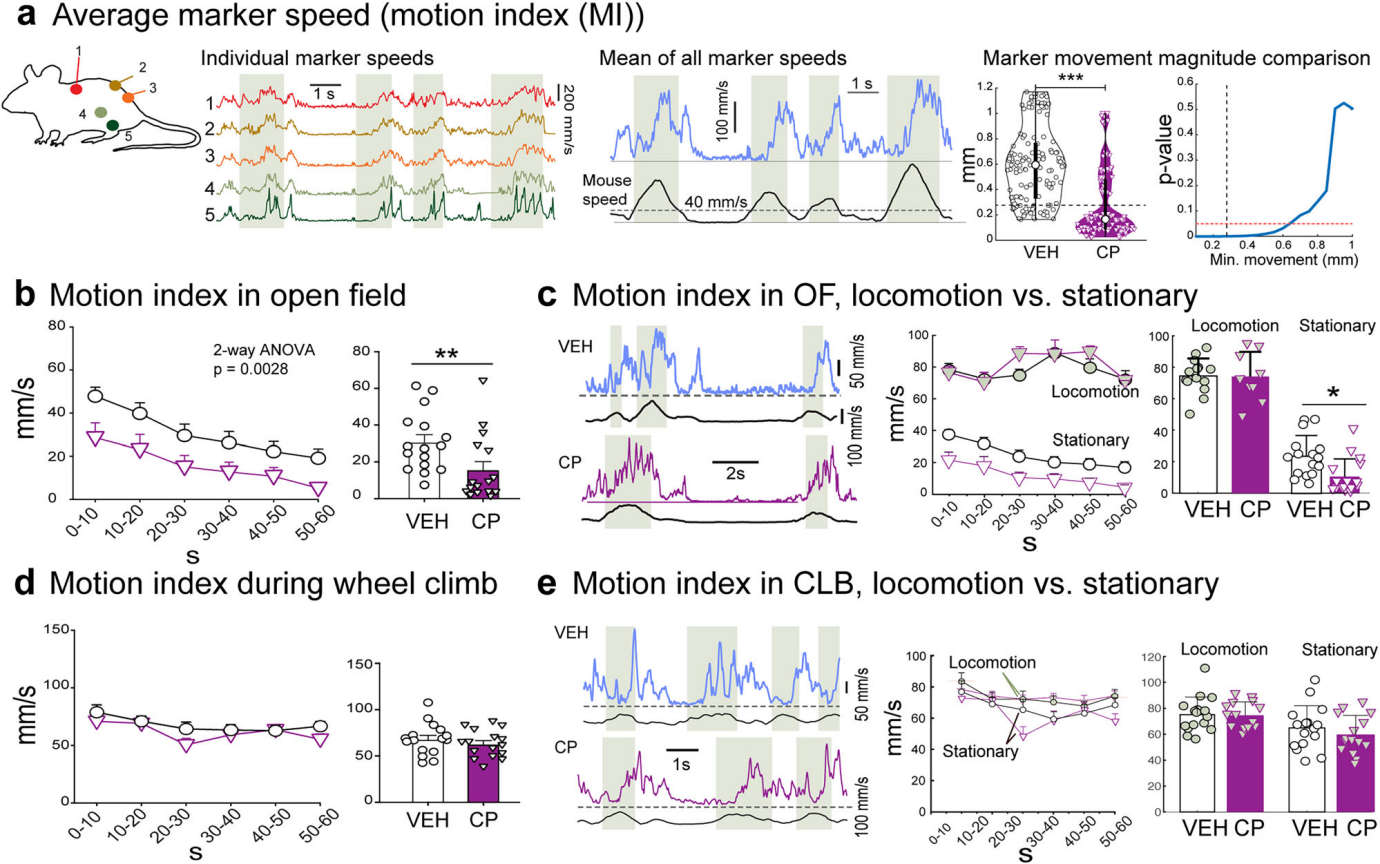
MI resolves fine effects of CP55,940 (CP). ***a***, Construction of MI as the average speed of all markers. Schematic of marker locations and their instantaneous speeds (left) and during a period consisting of locomotion (shaded areas) and immobility. Middle panel shows the average of all marker speeds over the same period (blue) and mouse speed (black). Rightmost panels compare the mean 2D magnitude of marker movement over 10 frames between vehicle (VEH) and CP groups (violin plots), as well as the effect of minimal detectable movement size on the distinction between the groups. Black dashed line denotes an estimated noise floor (0.28 mm) based on projecting our 3D triangulation residual mean of 0.35 to 2D. Red dashed line denotes *p* = 0.05. ***p < 0.001, Wilcoxon ranksum ***b***, MI during OF exploration. CP significantly decreases MI in 10 s bins (left) and in trial averages (left). **p < 0.01 in paired *t*-test. ***c***, In OF, CP decreases MI during immobility periods. Left panel shows example MI and speeds of a mouse in a period of two locomotory episodes and an intervening period of immobility for VEH (top) and CP (bottom) treated animals. Dashed line indicates MI = 0. Middle panel: binned MI values for periods of locomotion and stationarity. Right panel: whole-trial means for locomotion and immobility. *p < 0.05 post hoc comparison. ***d***, MI is not affected by CP during climbing (CLB), binned (left), or overall (right). ***e***, No change in MI for either locomotion or time spent immobile during CLB. Panels as in (c).

**Figure 5.**
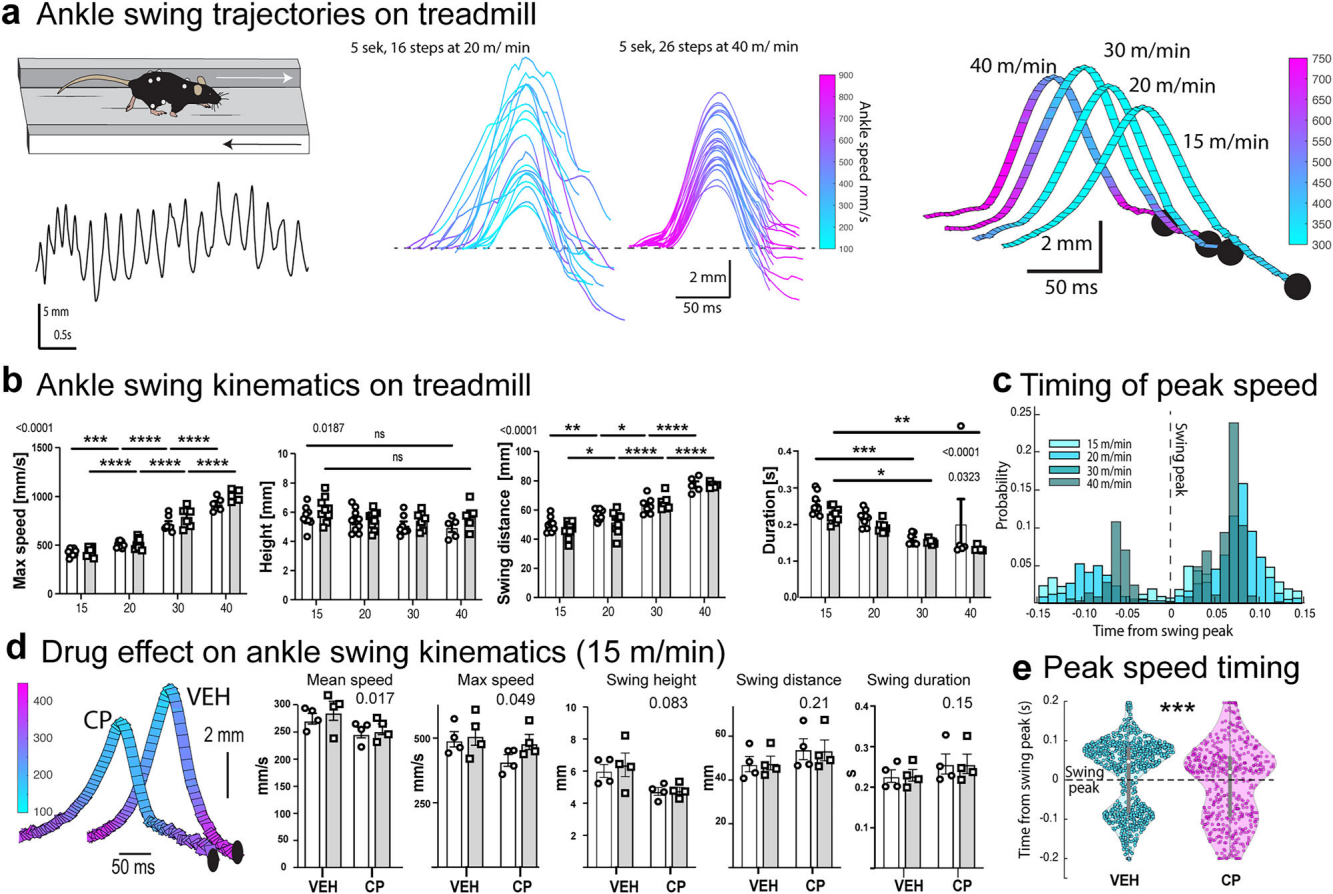
Hindlimb swing kinematics during treadmill running. ***a***, Left: schematic and a representative example of vertical ankle trajectory during continuous treadmill running at 20 m/min. Middle: individual swing trajectories color coded for instantaneous speed for 20 and 40 m/min running. Right: average ankle trajectories for swings during running at different speeds. Color indicates instantaneous speed. Trajectories are graphically arranged for visualization. ***b***, Ankle swing features for all mice running at all speeds. From left: maximal swing speed, swing height, horizontal swing distance, and swing duration. White and gray data represent left and right legs, respectively. Two-way ANOVA: factors speed *x* leg side—first number indicates *p* value of speed factor and second number *p* value of leg side factor. *p < 0.05, **p < 0.01, ***p < 0.001, ****p < 0.0001 post hoc comparison (*n* = 9). ***c***, Timing of highest ankle speeds with respect to vertical swing peak time (dashed line) shown for all speeds examined. Regardless of running speed, ankles move fastest when close to the treadmill. ***d***, Effect of CP55,940 (CP, 0.3 mg/kg) on swing kinematics. Leftmost panel shows average swing trajectories for a representative mouse in vehicle (VEH) and CP conditions. Color coding and graphical arrangement as in panel (a). Bar graphs show swing kinematics for 15 m/min running speed; white and gray data correspond to left and right legs. Two-way ANOVA: factors treatment *x* leg side (*n* = 6), *p* value indicates effect of treatment. ***e***, CP affects the timing of peak ankle speeds during 15 m/min treadmill running. ****p < 0.0001, Mann–Whitney U test.

**Figure 6.**
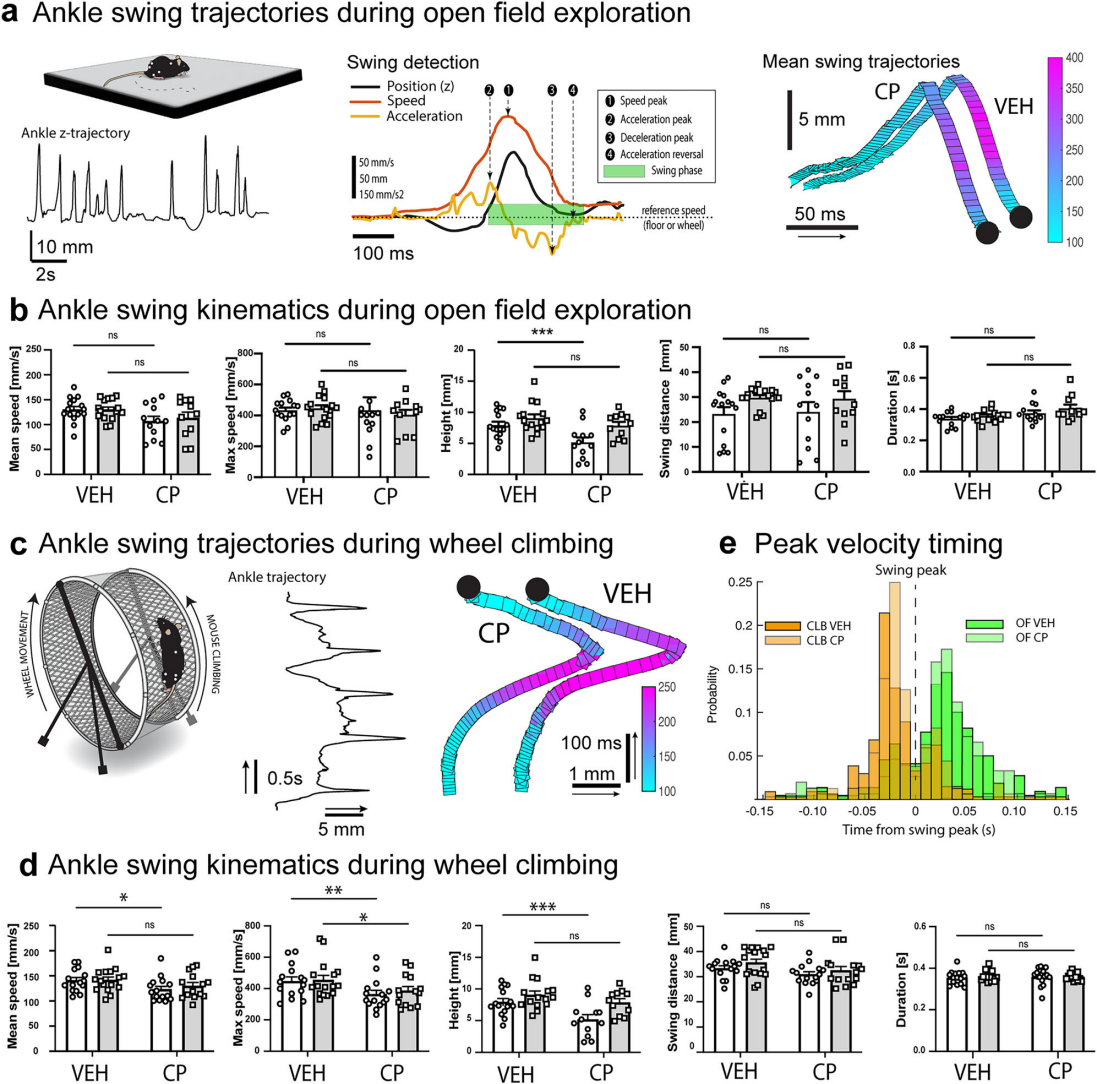
Hindlimb kinematics during intermittent locomotion. ***a***, Left: schematic and representative ankle trajectory during a period of voluntary OF exploration. Note variability of movements. Middle: schematic describing step detection in OF and climbing (CLB) tasks. Right: average ankle trajectories for a representative mouse exploring the OF in vehicle (VEH) and CP55,940 (CP) conditions. Color coding represents instantaneous ankle speed. Trajectories are arranged graphically for easy visualization. ***b***, Summary of ankle swing kinematics on OF under VEH and CP conditions. White and gray bars refer to data from left and right ankles, respectively. ***c***, Left: schematic and representative ankle trajectory during CLB, shown in plane perpendicular to wheel and corrected for wheel movement. Time axis runs upwards. Right: average ankle swing trajectories from a representative mouse under VEH and CP conditions, color coded for instantaneous speed. Time axis runs upwards. ***d***, Timing of ankle speeds with respect to swing peak timing differs in OF and CLB tasks (green vs orange bars). CP does not affect timing compared to VEH condition (weak vs strong colors). ***e***, Summary of ankle swing kinematic measures for VEH and CP conditions. White and gray data represent left and right ankle measurements, respectively. Two-way ANOVA, factors treatment *x* leg side: *p < 0.05, **p < 0.01, ***p < 0.001 in post hoc comparison (*n* = 16).

- General locomotion tracking (Fig. 3): The midpoint between the two hip markers was used as a reference point for tracking general locomotion in the arena. Frame-by-frame speed of this point was calculated based on the 3D displacement distance over 1 s. For CLB and TRM tasks, displacement was calculated relative to the surface movement, which was tracked using markers attached to the edges of the wheel and treadmill belt. To detect the onset of locomotion, the velocity of the hip center point was calculated using displacement over 100 frames (∼300 ms) for improved temporal resolution. “Locomotory episode” was defined as periods where the mouse moved faster than 40 mm/s for a minimum of 100 frames, allowing for brief dips below the threshold (up to 50 frames; ∼165 ms) which corresponds to directed fast speed walking (as opposed to slow stepping in place).
- *Motion index (MI; Fig. 4)* was calculated as the average speed of all markers, based on the 3D displacement distance over 10 frames (∼33 ms). This metric provided a comprehensive measure of whole-body movement, capturing both locomotory and non-locomotory activities.
- *Step detection (Figs. 5, 6, and 8)* relied on the vertical movement of the ankle marker during continuous locomotion during the TRM task. Swing periods were defined as the intervals between consecutive minima in the vertical trajectory. For OF and CLB tasks, swing definitions were based on ankle speeds calculated over 30-frame (∼100 ms) intervals. Swing start and end points were identified by detecting acceleration and deceleration peaks flanking high-speed ankle trajectories. This method addressed challenges arising from non-orthogonal ankle movements and was grounded in the principle that locomotory steps generate propulsion, which must involve a distinct acceleration event. During CLB and TRM tasks, ankle speed was calculated relative to surface motion, tracked using rim- or belt-attached markers.

### Kinematic step measure definitions

- *Duration* is measured from the start to the end of a swing period, with the endpoint defined as the conclusion of the ankle deceleration phase in OF and climbing.
- *Mean and maximum ankle speeds* are calculated for each ankle in each mouse by averaging the mean or maximum frame-by-frame speeds observed during swing periods.
- *Swing height* definition depends on the locomotion context. For horizontal locomotion (OF and TRM), swing height is the maximum vertical extent of the swing trajectory. During CLB task, swing height is defined as the amplitude along the second principal component of the 3D trajectory to account for the variable body angles around the wheel.
- *Swing distance* refers to the Euclidean distance between the ankle positions at the start and end of the swing.
- *Swing trajectory length* represents the total distance the ankle travels in 3D space between the start and end of the swing.

### Tremor analysis

Tremor analysis was conducted using Fourier decomposition to construct the power spectrum within the range of 6–18 Hz. To reduce noise, spline interpolation with an automatically selected smoothing factor was applied. Tremor boundaries were identified using two complementary strategies: (1) derivative noise analysis, with a threshold set at 10% of the maximum derivative value, and (2) detection of local maxima and minima if the derivative strategy was unsuccessful.

Tremor amplitude was quantified from trajectories filtered within the individual tremor frequency bands identified for each marker. The Hilbert transform was applied to compute the instantaneous amplitude from the modulus of the analytic signal. Then these amplitudes were averaged across windows, axes, and markers for statistical analysis.

Correlations between the instantaneous phases and amplitudes were derived from the Hilbert transform for all combinations of axes and markers. This analysis was used to identify the directional synchronization of oscillations within individual markers and across multiple markers.

### Data analysis

For grouped animal results, data are presented as mean + standard error (SEM) and analyzed using a one-way or two-way analysis of variance (ANOVA) or paired *t*-student test as appropriate. We did not exclude any data. In case of missing values, data were analyzed by fitting a mixed model, rather than by repeated measures ANOVA. Dunnett’s or Tukey post hoc comparisons were used. To examine the overall performance of motion capture irrespective of the variability between animals (Fig. 2), we pooled all frames and markers across all animals, separating the data into locomotory and non-locomotory frames. We used the non-parametric Mann–Whitney U test (ranksum) for comparisons because the data did not meet normality assumptions. Differences were considered significant at the level of *p* < 0.05. Statistical analysis was performed with GraphPad Prism version 9.00 or Matlab (ver. 2024a, Mathworks) ran on MacOS Sonoma 14.4.1.

## Results

### Realization of reliable long-term motion capture tracking in mice

Figure 1 shows the arrangement of the motion capture recording environment [(a) and (b): inset shows a close-up of a mouse wearing the retroreflective markers during a recording session], our chosen marker placements on a mouse, and an example frame with 3D reconstruction of the markers [(c): Movie 1]. Six Qualisys Oqus 7+ cameras (Qualisys) are positioned around the experimental arena with a slight downward tilt and a 60 ° angle between each neighboring camera. Notably, adding more cameras does not benefit tracking performance unless there are occlusions caused by task-specific equipment or image acquisition parameters are set suboptimally. Importantly, as the motion capture cameras only generate marker trajectories by means of on-board coordinate triangulation without saving video images, an video camera (Miqus Hybrid, Qualisys; 85 fps) is synchronized with the motion capture system to provide a conventional record of the experiment. Mice are thoroughly habituated to the experimental room and arena to minimize the effects of stress on behavior (see Methods, Behavioral training).

**Movie 1.** Raw 3D reconstruction of marker positions during OF exploration shown in real time. Red color of a marker indicates break in automatic tracking. [View online]

The foundation of the marker implant is a barbell-shaped stainless steel piercing with screw-on spheres, originally intended for human skin decoration purposes. 18–20G gauge piercings with 6, 7, and 8-mm-long shafts are used as appropriate for each body part. The shafts weigh between 55.18 ± 0.26 mg (6 mm) and 69.38 ± 0.26 mg (8 mm); the bare screw-on spheres weigh 94.54 ± 1.52 mg. For preparing markerheads for motion capture use, the spheres (Fig. 1*d*) are covered with thin retroreflective tape strips and polyethylene film, before coating them with liquid ultraviolet light-curable plastic (final weight of retroreflective sphere: 239.37 ± 2.43 mg; see Methods for full construction details). During the implantation procedure performed under isoflurane anesthesia, small holes are punctured at key locations on mouse skin to allow subcutaneous threading of the barbell piercing. The weight of the 10 uncoated piercings worn by mice daily in this study was 1.2–1.3 g, amounting to not more than 5–7% of mouse body weight. The natural process of skin healing leads to secure attachment of markers for extended use, and marker loss does not occur within the first 3 months after implantation.

At the beginning of each recording, the steel spheres are exchanged for the larger retroreflective markerheads. As the animals are thoroughly habituated to behavior while wearing the larger markerheads, they do not display any discomfort. Notably, while the total weight of the retroreflective markers (approx. 3 g) can reach 12% of the mass of a 25 g mouse, the distribution of the weight across body parts suggests the effort is not greater than that of many commonly used head-mounted miniature microscopes (de Groot et al., 2020).

### Performance of MBMC performance in freely moving mice

An ideal motion capture methodology would allow precise, accurate, and robust tracking of desired anatomical keypoints over extended periods (weeks) during naturalistic, three-dimensional behavior with no need for extensive post-processing or missing data imputation. The performance of the capture should be consistent regardless of the position of the subject within the recording volume (e.g., distance to the recording area borders) or behavioral state.

To validate the function of our motion capture system within these parameters, we used a dataset that consisted of a cohort of 10 adult mice, wearing 10 markers, exploring a square OF arena for 1 min (three trials on different days; recorded at 300 fps, resulting in total of 5400,000 data points). Figure 2*a* shows the vertical trajectories of all markers on the left side in a representative example of a complete recording, without any post-processing besides trajectory labeling in the Qualisys QTM software. As seen in the segment shown extended in Figure 2*b*, the gaps in trajectories were short and mostly appeared in a single trajectory at a time. The tracking reliability was consistently high (more than 80% markers were tracked in 95% of frames; Fig. 2*c*). We noted that during passive immobility, mice often sit in a posture that partly occludes leg and shoulder blade markers leading to slightly more frequently missing trajectories (median fraction of visible markers per frame 0.97 for both locomotory and non-locomotory frames, but 95th percentile 0.82 and 0.77 for locomotory and non-locomotory frames, respectively; *p* = 0.012, Mann–Whitney U test). However, especially during locomotion, long gaps were rare [median gap duration 22 and 27 frames (∼73 and 90 ms); 95th percentile of gap durations 184 and 289 frames (∼607 and 954 ms), in locomotory and non-locomotory frames, respectively; *p* < 0.001 (Mann–Whitney U test); Fig. 2*d*] and were contributed mainly by a single missing marker (Fig. 2*e*, left). Naturally, individual markers can become less visible if their surface degrades leading to a situation where they can be less perfectly tracked throughout the trial. These individual cases are easily identifiable as “paths” of poor tracking when examining the spatial distribution of gaps during the experiment (Fig. 2*e*, right).

A standard way of quantifying tracking precision is built on the residual triangulation value, a measure of how precisely the 3D position of each marker can be reconstructed (“triangulated”; Hartley, 2003) from multiple camera views. As each camera captures a 2D projection of the marker, triangulation combines these multiple 2D images to estimate markers’ 3D positions. The residual of triangulation represents the discrepancy or error between the reconstructed 3D position and the underlying 2D projections. In our six-camera setup, these triangulation residuals ranged between 0.2 and 0.6 mm during experimental recordings, with slightly lower performance during locomotion [median residual 0.36 and 0.35 mm; 95% percentile bounds 0.53 and 0.51 mm, for locomotory and non-locomotory frames, respectively; *p* < 0.001 (Mann–Whitney U test); Fig. 2*f*].

Furthermore, leveraging the fact that our marker pairs are placed at known distances between each other, we could estimate the real tracking accuracy that depends on the size, visibility, and sphericity of the markers. Across all markers, the discrepancy in the tracked distance between the pair of markers and their known distances was also in the submillimeter range [median error 0.35 and 0.37 mm; 95% percentile bounds 0.63 and 0.64 mm for locomotory and non-locomotory frames; *p* < 0.001 (Mann–Whitney U test); Fig. 2*g*]. These measurements indicate that even when used in the challenging context of a moving mouse with hand-made markers, it is reasonable to have confidence in submillimeter location accuracy not far from the device capability reported for best-case scenarios (Topley and Richards, 2020).

In markerless tracking approaches, small high-frequency positional fluctuations (“jitter”) arising from ambiguity among nearby pixels can complicate the analysis of subtle behaviors, as these fluctuations could be misinterpreted as distinct events (Weinreb et al., 2024). Although they can to an extent be removed from trajectory data in post-processing, such cleaning may inadvertently remove real features of movement. In contrast, the marker-based trajectories we recorded rarely exhibited such jitter, especially under well-calibrated conditions where markers remain visible from multiple camera angles. As exemplified by the raw trajectories recorded during passive immobility periods (Fig. 2*h*), jitter was minimal. Instead, the predominant subtle movement observed was a low-amplitude (∼8 Hz) oscillation of shoulder blade markers, possibly reflecting respiration (Fig. 2*i*, top trace). Ankle markers occasionally showed single-frame glitches of less than 100 μm (Fig. 2*i*, bottom trace). Such minor artifacts are straightforward to remove by simple interpolation, similarly to the handling of short gaps.

Taken together, these assessments indicate that marker-based tracking on freely moving mice can offer continuous, submillimeter precision tracking of anatomically relevant markers with minimal data loss or jitter. This allows for confident use of its output in subsequent analyses without further post-processing such as smoothing or model fitting.

### Context-dependent behavioral disruption of general locomotory parameters by cannabinoid receptor agonist

To validate our method compared to established behavioral assays, our objective was first to reproduce well-characterized behavioral alterations induced by CP55,940 (CP; 0.3 mg/kg), a cannabinoid CB_1_ and CB_2_ receptor agonist. Although tracking general parameters such as animal position and speed can be adequately accomplished with markerless or even simpler image-thresholding-based analysis [such as Bonsai-RX (https://bonsai-rx.org/ Lopes et al., 2015) and Ethovision XT (Noldus; https://noldus.com/ethovision-xt)), demonstrating these effects under our experimental conditions ensures that marker implantation and lack of enclosure in the motion capture setup do not obfuscate the well-established but subtle effect. To this end, we assessed voluntary locomotion during OF exploration, and further added two novel locomotory tasks: CLB and TRM (see examples in Movies 2, 3, and 4). As illustrated in Figure 3*a*, we monitored the position and instantaneous speed of the midpoint of the hip markers to quantify the total distance traveled during a trial (Fig. 3*b*), time spent locomoting (Fig. 3*c*), and locomotion speed (Fig. 3*d*). Indeed, as expected and previously demonstrated for the moderate dose of CP using conventional behavioral apparatuses (Patel and Hillard, 2001; Ignatowska-Jankowska et al., 2015a), mice exhibited a slight inhibition of locomotion expressed as a trend toward decreased distance traveled [*F* (1, 15) = 4.122, *p* = 0.060] and significantly decreased time spent locomoting [*F* (1, 15) = 6.824, *p* = 0.0196]. There was no effect on locomotion speed [*F* (1, 23) = 0.02, *p* = 0.89]. Surprisingly, locomotion during climbing was completely unaffected—distance traveled (*p* = 0.31), time spent locomoting (*p* = 0.51), and speed (*p* = 0.94) were not altered by CP administration, although OF and CLB tasks were carried out within minutes of each other. Interestingly, CP significantly decreased the maximum speed at which mice were able to run on a motorized treadmill, on average by 10 m/min, from 28 ± 3.7 to 15 ± 3.9 (mean ± SEM; *t* = 4.33, *p* = 0.012).

**Movie 2.** Video and motion capture reconstructions of full 1 min trials involving OF exploration in the same mouse treated with vehicle and CP55,940. [View online]

**Movie 3.** Video and motion capture reconstructions of full 1 min trials involving voluntary wheel climbing in the same mouse treated with vehicle and CP55,940. [View online]

**Movie 4.** Video and motion capture reconstructions of full 30 s trials involving running on a motorized treadmill, in the same mouse treated with vehicle and CP55,940. Two highest speeds this mouse reached are shown for both conditions. [View online]

### MBMC provides additional resolution to activity monitoring

Although basic parameters such as mouse position and average speed within a 2D environment—or even distance traveled on a climbing wheel—can be adequately captured using simpler means, additional insights are gained through MBMC when assessing small-amplitude movements across multiple markers.

As illustrated in Figure 4*a*, we define a “MI” as the average instantaneous speed of all markers, providing a sensitive measure of subtle body movements. Naturally, MI increases during locomotion and scales with the animal’s speed. Importantly, MI also captures very small movements occurring during stationary periods, such as grooming, sniffing, or minute postural adjustments, which can be informative of the animal’s behavioral state beyond overt locomotion alone (Kalueff et al., 2016). Importantly, movements during stationary periods were extraordinarily small [mean marker displacement over 10 frames (33 ms) in VEH: 0.6 ± 0.3 mm; CP: 0.29 ± 0.27 mm; *p* < 0.001, Wilcoxon rank-sum] and their reliable detection is necessary for differentiating between groups.

Indeed, CP-treated mice exhibited consistently lower MI values throughout OF trials ([*F* (1, 15) = 12.71], *p* = 0.0028; Fig. 4*b*). This decrease in MI was entirely driven by suppression of movements during stationary periods (Fig. 4*c*; *p* = 0.025 and 0.54 for changes in average stationary and locomotory MI, respectively), consistent with unchanged locomotion speeds. In contrast, MI during the CLB task did not differ significantly between CP- and vehicle-treated trials overall (*p* = 0.27; Fig. 4*d*), nor when comparing stationary and locomotory periods separately (*p* = 0.22 and 0.94; Fig. 4*e*). This reflects the tendency of mice to move end shift posture constantly even during the brief climbing pauses (Fig. 4; Movie 3).

### Ankle swing kinematics during fast treadmill running

Moving beyond the description of general whole-body locomotion parameters, we examined the 3D trajectories of hindlimb ankles during running on a treadmill at different speeds (Movies 5–7 for reconstructions with real time and slowed-down framerates) to see if we could identify features specifically affected by CP administration. As shown in Figure 5, *a* and *b*, individual steps were readily identifiable with relatively uniform waveforms that, with increasing treadmill speeds, decreased in amplitude but covered longer horizontal distances due to faster movements of the limbs. Importantly, during slower running (15 m/min), the ankle moved at relatively uniform speed, but faster running was associated with an increasingly sharp timing of the peak ankle speed to match the contact with the treadmill (Fig. 5*c*) and the ankle slowed during the swing peak and the downward swing phases.

**Movie 5.** Close-up video and motion capture reconstruction for two segments of high-speed running on a treadmill. [View online]

**Movie 6.** Video and motion capture reconstructions of a full 30 s trial of a single mouse running on a treadmill at various speeds. [View online]

**Movie 7.** Close-up motion capture reconstruction of a segment of high-speed running (40 m/min treadmill speed), in normal speed and slowed-down to 20%. [View online]

In line with our observation that the mice were unable to run at high speeds after CP administration, we found that their swing kinematics were also affected (Fig. 5*d*). Comparison of kinematic measures between vehicle- and CP-treated mice running at the lowest tested speed (15 m/min, the only speed CP-treated mice reliably ran) revealed significantly decreased ankle swing height [*F* (1, 3) = 39.00, *p* = 0.0083] as well as a decrease in mean [*F* (1, 3) = 22.96, *p* = 0.017) and maximum *F* (1, 3) = 10.26, *p* = 0.049] ankle swing speed. There were no changes in the swing distance (*p* = 0.20). Intriguingly, we noticed a slight but significant change in the distribution of peak ankle speed timing so that the down-swing speed peak shifts closer to the swing peak (median peak speed timing 0.08 and 0.06 s after swing peak for vehicle and CP groups; *p* < 0.0001, Mann–Whitney U test; Fig. 5*e*).

### Ankle kinematics during voluntary locomotion in the OF

Behavioral models commonly used to examine mouse limb kinematics include forced locomotion on a treadmill (Ueno and Yamashita, 2011; Lemieux et al., 2016) or in narrow walkways (Ueno and Yamashita, 2011; Lemieux et al., 2016). Restricting movement into one direction greatly simplifies motion tracking and allows collecting supposedly uniform step trajectories during a short experimental trial. However, the emotional and motivational state of the mouse can modulate motor behavior (Braine and Georges, 2023), and the kinematic characteristics important for self-driven voluntary locomotion might be masked if locomotion is forced or restricted.

Thus, since we had observed that CP caused a subtle suppression of locomotor activity in the OF (Fig. 3), we wondered if the kinematics of the OF steps would be affected similarly to those seen in the treadmill trials (decrease in speed and swing height).

Voluntary exploratory locomotion in mice is inherently variable and consists of periods of forward movement (locomotory episodes) and intermittent periods of other activities such as grooming or postural changes. Importantly, many of such nonlocomotor behaviors involve limb movements, and as a result it is not possible to reliably detect locomotor steps by vertical motion alone. Therefore, we constructed a more context-appropriate locomotory step detection procedure (Fig. 6*a*, left and middle panels). Briefly, we delineate locomotory ankle swing periods by acceleratory and deceleratory events rather than position. As shown for an example animal in Figure 6*a* (rightmost panel), exploratory ankle swings were characterized by a sloping upward motion followed by a rapid ankle drop, resulting in an asymmetric trajectory that was surprisingly unaffected by the administration of CP. Examination of the kinematic parameters of the swing in all animals (Fig. 6*b*) confirmed the observation that a moderate dose of cannabinoid agonist did not lead to a decrease in the speed, distance, or duration of the ankle swing. However, we noticed a decrease in the average heights of the left ankle swings, possibly reflecting a drug-induced bias in behavioral lateralization.

Thus, we conclude that the slight locomotory inhibition in the OF task induced by CP is mostly expressed as a suppression of activity during non-locomotory periods and somewhat increased reluctance to move, with very limited effects on locomotory kinematics.

### Ankle kinematics during voluntary wheel climb locomotion

Comparison of general locomotion parameters during OF and CLB tasks under CP treatment revealed a noteworthy and novel finding: there was no suppression of locomotion in CLB task (Fig. 3). To examine whether ankle kinematics were affected by CP during CLB, we detected ankle swings using the same criteria as for OF, with the difference that speed and acceleration were calculated with respect to the wheel movement, tracked using markers attached to the rim. Furthermore, the swing “height” was defined perpendicularly to the wheel (Fig. 6*c*). Given that none of the general locomotion parameters were affected during CLB, we were surprised to find that mean [*F* (1, 15) = 7.66, *p* = 0.014] and maximum [*F* (1, 17) = 15.66, *p* = 0.001] ankle speeds decreased significantly in the CP-treated group (Fig. 6*d*) compared to vehicle. Furthermore, the swing height [*F* (1, 15) = 27.17, *p* = 0.0001] was significantly reduced, asymmetrically in the left leg [*F* (1, 15) = 16.56, *p* = 0.001], as was also observed in the OF and treadmill data (Fig. 5). There was a small decrease in swing distance [*F* (1, 16) = 4.78, *p* = 0.044] but duration of the swing was not affected [*F* (1, 15) = 0.025, *p* = 0.87].

Finally, we compared the timing of peak ankle speed with respect to the ankle swing period during horizontal or vertical voluntary locomotion, as was done for treadmill locomotion (Fig. 6*e*). Although CP did not alter ankle speed timing in OF or CLB tasks, we found a clear difference between the three locomotor contexts. First, in contrast to the sharp timing of the high-speed motion at the onset of the swing on the treadmill, the fastest ankle movements occurred just after and before the swing peak for OF and CLB, respectively. These differences reinforce the notion that even though all three contexts involve locomotion, they may involve distinct motor programs of the limb, potentially leading to different responses to pharmacological interventions.

### Harmaline tremor

Going beyond what previously has been possible in the realms of mouse kinematic tracking, we aimed to test whether MBMC could be used to investigate very fine movements such as tremor. Pathological tremor is a symptom of Parkinson’s disease (PD; Helmich et al., 2013) and analysis and decomposition of its kinematic characteristics using accelerometer data from wearables or even smartphones (Duque et al., 2020; Fujikawa et al., 2022) show promise for the diagnostic process for differentiating PD (Rahimi et al., 2015; Angelini et al., 2024) from essential tremor (ET; Welton et al., 2021). Despite the clear need for fine measurement of tremor in animal models, current approaches are rather crude and limited to methods such as quantifying tremor frequency band fluctuations reported by force plates (Wang, 2022). Among others, questions related to body part specificity and tremor lateralization in animal models have not been possible to investigate.

**Figure 7.**
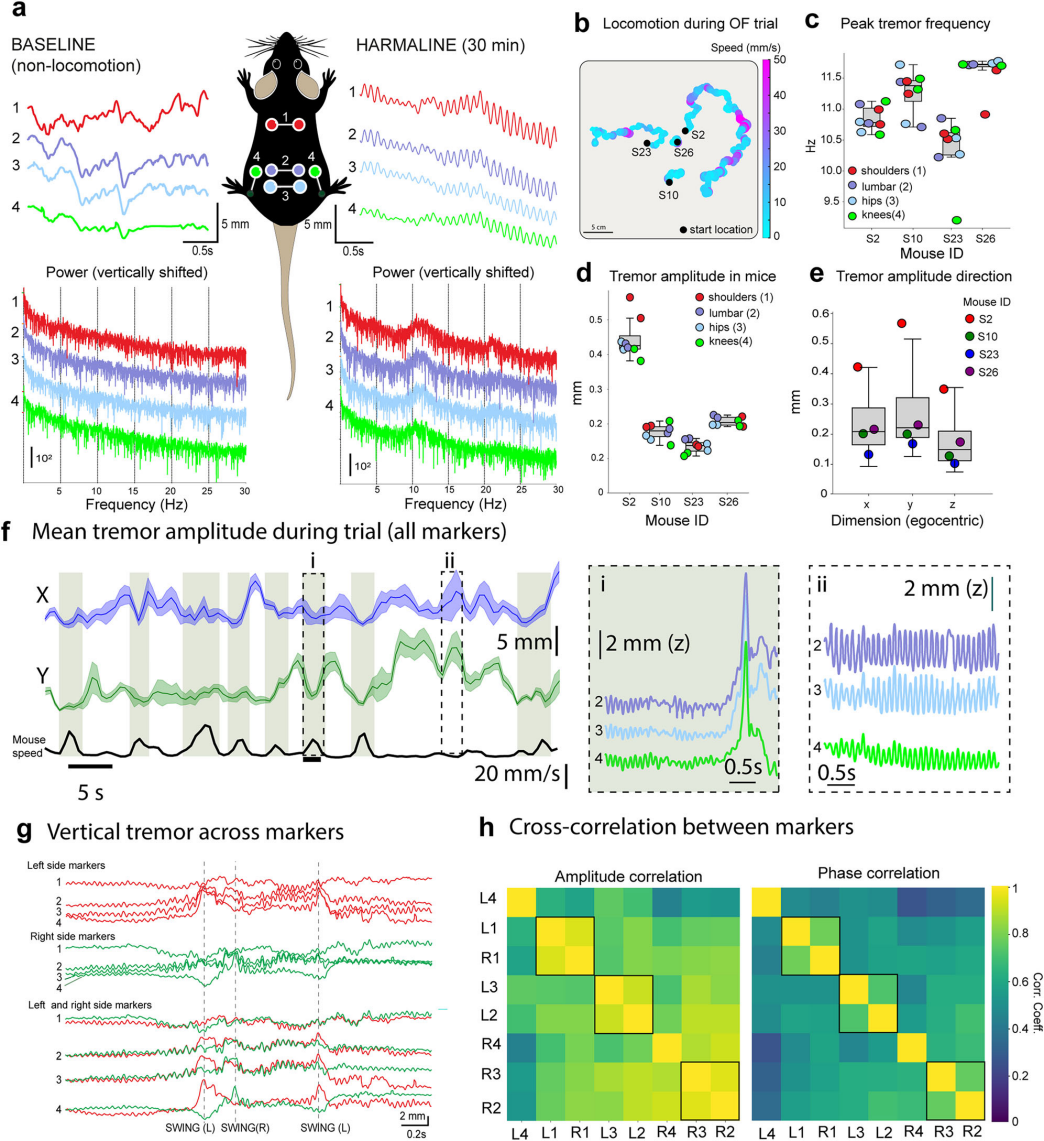
Motion capture of full-body harmaline tremors. ***a***, Unilateral, vertical trajectories of back, hip, lumbar, and knee markers from a mouse before (left) and after harmaline administration, during a period of passive immobility. Bottom panels show power spectrum densities for the same markers; the traces are vertically shifted to easier visualization. ***b***, Movement in the OF arena of the four mice examined after harmaline administration. Color coding indicates locomotion speed. ***c***, Peak tremor frequency for individual markers (colored symbols) on the four mice (box plots). ***d***, Mean tremor amplitude for individual markers (colored symbols) on the four mice (box plots). ***e***, Mean tremor amplitude for individual mice (colored symbols) in the three dimensions (box plots, in egocentric coordinates). ***f***, Tremor amplitude in *x* and *y* dimensions, calculated over a time window of 150 frames (∼500 ms). Black trace shows mouse speed. Shaded green rectangles indicate periods of movement faster than 10 mm/s. Panels (i) and (ii) show the vertical trajectories of hip, lumbar, and knee markers during preparation to a step (i) and passive immobility (ii). ***g***, Vertical trajectories of the markers during a sequence of steps. Traces on top are arranged by side on top, vertically aligned at the peak of the first swing (dashed lines). Bottom traces are arranged by marker position to highlight precise phase-locking. Traces are vertically aligned at first swing start. ***h***, Amplitude (left) and phase (right) cross-correlation matrices for shoulder blades, lumbar spine, hip, and knee markers in all four animals [indicated by numbers as in panel (a)]. L and R refer to left and right sides. Rectangles highlight three pairs of markers with high correlation.

To examine the possibility of using MBMC for decomposing body tremors across body parts, we tracked four pairs of markers (shoulder blades, lumbar spine, hips, and knees) in four mice freely behaving in the OF after administration of tremorgenic harmaline (20 mg/kg; Handforth, 2012). Due to the posture and very low mobility of the harmaline-treated animals, ankle markers were often not visible and were not tracked in this analysis. As shown in Figure 7*a*, the tremors are visible in all tracked markers (top panels) with a clearly identifiable peak at “classic” frequencies (8–12 Hz; Milner et al., 1995; Martin et al., 2005; Paterson et al., 2009; Woodward et al., 2022; Movie 8). All mice displayed severe suppression of locomotion (Fig. 7*b*), so that two of them (S10 and S23) did not take any forward steps during the recording despite exhibiting other stationary behaviors such as grooming. The mean peak of the tremor frequency varied more between animals than among the markers on a single animals (ANOVA values for markers in each animal, *F* = 0.181 − 5.41, *p* = 0.01 − 0.84; comparing the four animals in the experiment, *F* = 18.61, *p* < 0.0001; Fig. 7*b*). Amplitude of the tremor varied between markers on an animal likely reflecting differences in movement range, and was overall higher in the individual that was locomoting most (S2; Fig. 7*d*; ANOVA for markers in each animal, *F* = 7.65 − 22.14; for the four animals in the experiment, *F* = 138.03, *p* < 0.0001). Across animals, the vertical-directed movement of tremor was slightly but insignificantly smaller than horizontal (Fig. 7*e*; *F* = 0.51, *p* = 0.62).

**Movie 8.** Close-up motion capture reconstruction of raw, unprocessed marker positions during a full 1 min recording of a mouse under harmaline influence. Text in upper right corner indicates gaps in marker tracking. [View online]

The higher tremor amplitude in the mouse that was moving the most prompted us to investigate whether the tremor would be specifically enhanced during periods of increased activity. Figure 7*f* shows data from mouse S2 that showed periods of motility. The movement was always slow, never reaching our conventional forward displacement threshold of 40 mm/s as a definition of locomotion, and the locomotor episodes (>10 mm/s; indicated by green shading in Fig. 7*f*) consisted of not more than 2–3 steps at most. However, in this individual, the tremor was in fact suppressed during forward movement and reached highest amplitudes during periods of stillness [insets (i) and (ii) in Fig. 7*f* showing vertical trajectories of markers lumbar, hip, and knee].

Next, we examined to what extent the tremors are correlated across the different parts of the body. To our surprise, we found a very clear phase and amplitude correlation, not only between the left and right sides of the animal that could be explained to some extent by the physical connection between left and right markers (Fig. 7*g*, top traces) but also along the entire rostro-caudal “chain” of markers (from shoulders to knees; Fig. 7*g*, bottom traces). In fact, in the four animals, a positive correlation was found in both amplitude (Fig. 7*h*, left) and phase (Fig. 7*h*, right) for all markers, including distant pairs (e.g., left shoulder vs right hip). The whole-body correlation of tremor is particularly well visible in slow-motion videos of tremoring animals (Movie 9).

**Movie 9.** Close-up motion capture reconstruction of raw, unprocessed marker positions in a mouse under harmaline influence, shown slowed down to 20%. Text in upper right corner indicates gaps in marker tracking. [View online]

### Ankle kinematics after harmaline administration

**Figure 8.**
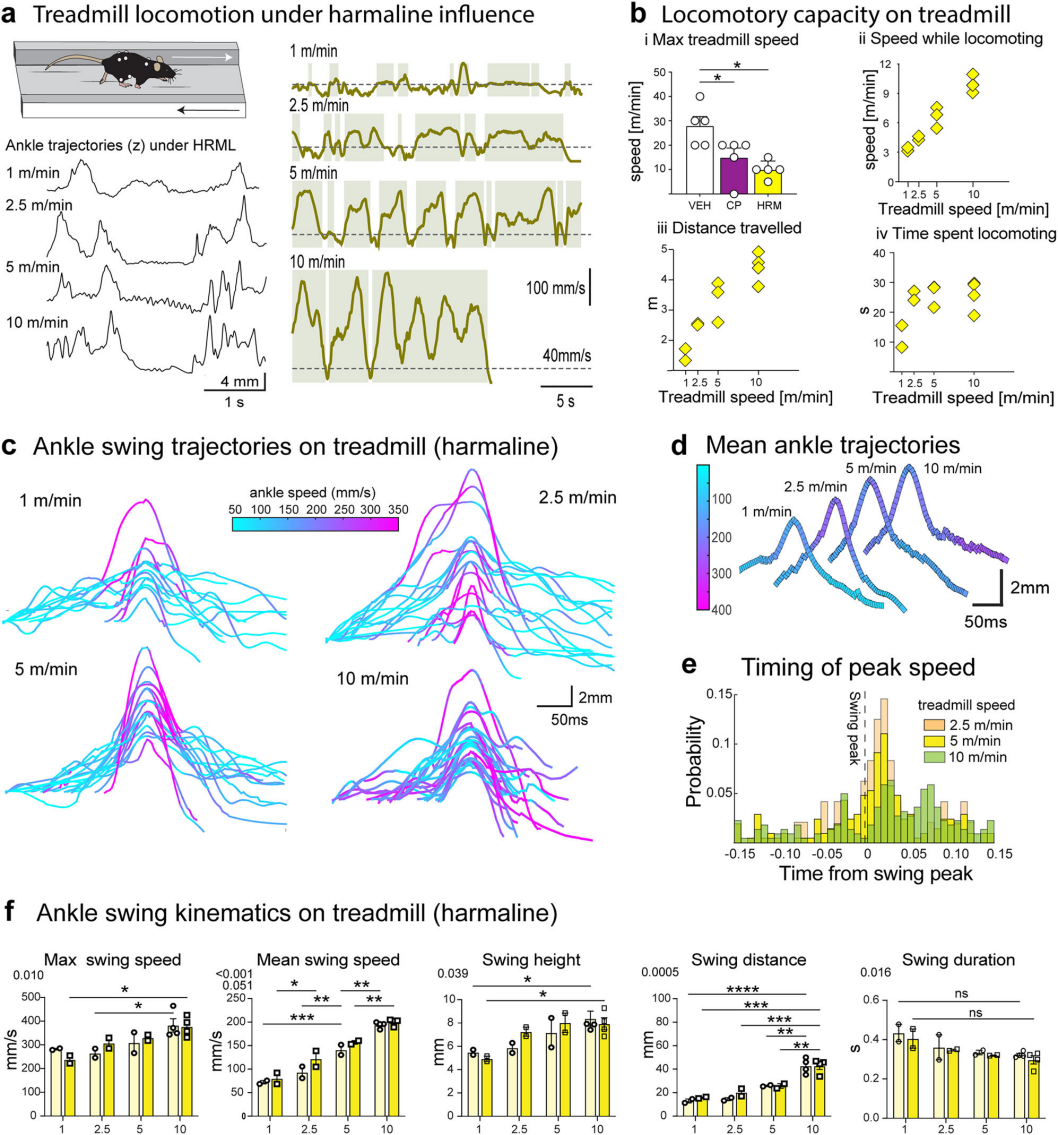
Ankle swing kinematics during treadmill locomotion under harmaline-induced tremor. ***a***, Left: schematic and representative vertical trajectories of ankle during running on a treadmill at different speeds. Right: mouse speed during full 30 s trials. Green shaded areas indicate periods of faster than 40 mm/s, indicated by dashed line. During fastest speed, the mouse was not able to continue locomoting for the full trial. ***b***, Locomotory capacity on treadmill. *i*, Comparison maximal speeds reached by mice in vehicle (VEH), CP55,940 (CP), and harmaline (HRM) conditions; each marker represents a single mouse. *ii*–*iv*, General locomotory parameters on treadmill at 1–10 m/min speeds. ***c***, Example vertical trajectories of ankle swings at different speeds from a single mouse. Color indicates instantaneous ankle speed. ***d***, Mean ankle trajectories for the same mouse. Color indicates instantaneous ankle speed. Traces are arranged graphically for visualization. ***e***, Timing of peak ankle speed with respect to ankle swing peak (dashed line) with the three highest speeds. ***f***, Summary of ankle swing kinematic measures for all animals during treadmill locomotion. Light and dark-colored data correspond to left and right ankles, respectively. Two-way ANOVA, factors speed *x* leg side: *p < 0.05, **p < 0.01, ***p < 0.001, ****p < 0.0001 in post hoc comparison (*n* = 6).

As the harmaline-treated mice did not locomote sufficiently in the OF nor on the climbing wheel (Movies 10 and 11), we examined limb kinematics on treadmill on which the mice were able to maintain instantaneous advancing speeds over 40 mm/s on up to 10 m/min treadmill speed (Fig. 8*a*; Movie 12). Even though their capacity for locomotion was significantly lower than that of the same animals without drug administration or with CP administration [Fig. 8*b*(*i*)–(*ii*)], the results suggest that the lack of locomotion in OF under harmaline could be caused by aversion to movement rather than a fundamental inability to locomote. Nevertheless, some of the harmaline-treated mice were unable to continue locomotion for the entire duration of the 30-s trial [Fig. 8*b*(*iii*)–(*iv*)], suggesting that exercise was unusually exhausting.

**Movie 10.** Video and motion capture reconstructions of full 1 min trials involving OF exploration in the same mouse treated with vehicle and harmaline. [View online]

**Movie 11.** Video and motion capture reconstructions of full 1 min trials involving wheel climbing in the same mouse treated with vehicle and harmaline. [View online]

**Movie 12.** Video and motion capture reconstructions of a full 30 s trial of a single mouse locomoting on treadmill at various speeds under harmaline influence. [View online]

The hindlimb trajectories of harmaline-treated mice showed clear oscillations before and after swing movements (Fig. 8*c*). Comparison with control groups was not feasible due to the refusal of untreated mice to maintain steady locomotion on treadmill speeds below 15 m/min. However, we noticed that the ankle movements leading to swing were very slow (cyan coloring in trajectories in Fig. 8*c,d*; Bologna et al., 2020b, 2020a). Furthermore, while maximum ankle speeds occurred robustly during the swing phase when locomoting on slow treadmill (1–5 m/min), at the highest speed (10 m/min) the timing of peak ankle speed was spread throughout the swing phase (Fig. 8*e*). This was in contrast to the timing of the maximum ankle speed in the control treadmill trials (Fig. 5*c*), possibly indicating that the tremor interferes with fine limb control during locomotion. This disruption of ankle speed timing aligns with the observation that mice could not increase ankle speeds or heights when the treadmill speed increased from 5 m/min to 10 m/min (Fig. 8*f*), which could lead to inefficient locomotion and eventual trial failure.

## Discussion

In this study, we establish that MBMC can track full-body kinematics in freely moving mice with submillimeter accuracy. By carefully optimizing marker construction, placement, and camera positioning, we obtained high-resolution trajectories that accurately capture both fine- and large-scale movements without the need for extensive post-processing. This approach complements markerless technologies, which excel in situations where marker attachment is infeasible (e.g., wild animals), high-throughput recordings are required, or pose estimates provide sufficient insight.

Importantly, markerless methods are inherently constrained to tracking movements that align with their training data, possibly limiting their ability to uncover novel features even under constant data acquisition conditions. In contrast, MBMC directly measures marker positions, generating data independent of prior models or datasets, enabling the detection of previously unknown motion patterns. This independence eliminates the need for training datasets or model retraining, making MBMC particularly valuable for applications requiring the reliable capture of subtle motions, such as tremor, or the precise quantification of low activity levels (e.g., the “MI” in this study).

In the following, we first address key methodological considerations and then discuss how our proof-of-concept pharmacological experiments illustrate the potential of MBMC to detect motor alterations in different locomotory contexts.

### Realization of marker-based mouse motion capture using skin-implantable markers

A core challenge with markerless motion tracking is its reliance on fully annotated training data sets and its susceptibility to occlusions, variable lighting, and generalization issues across individual animal differences. Marker-based tracking avoids these limitations by removing the need for background subtraction and explicitly defining the points to be tracked. This removal of ambiguity directly yields clean, ready-to-use 3D data, eliminating the need for complex and largely opaque post-processing pipelines.

In theory, the quality of MBMC data is entirely determined by the physical dimensions of markers and the optical characteristics of cameras. Thus, in the absence of noise, the process of reconstructing the marker positions by triangulation is straightforward (Hartley and Sturm, 1997; Kanatani et al., 2008). However, achieving such a level of tracking in mice presents unique challenges, primarily due to their tendency to destroy or remove foreign objects on their skin. This limitation has confined marker-based studies in mice to brief recordings, requiring frequent marker replacement, which can lead to inconsistent marker positioning and unnecessary stress to the animal. Furthermore, the looseness of mouse skin complicates accurate tracking of the underlying body structures using skin-top markers (Monsees et al., 2022). Although these issues are somewhat less pronounced in larger rodents, such as rats (Mimica et al., 2018; Marshall et al., 2021), mice remain the most widely used vertebrate models in systems and behavioral neuroscience (Ellenbroek and Youn, 2016), underscoring the need to develop mouse-appropriate solutions.

Our key innovation addresses these challenges through the use of three-part, under-skin implants with replaceable reflective heads. Installed under brief isoflurane anesthesia, these stainless steel implants anchor firmly as the skin heals. Mice resume normal behavior within a day and show no signs of discomfort. Over weeks to months, the implants remain stable without replacement. Although mice clean the implants as part of their grooming routine, they do not remove them. Before experimental recording, the stainless steel spheres capping the piercing shafts are substituted with retroreflective markers using a simple screw-on mechanism. This process does not require anesthesia and ensures consistently high-quality marker condition, resulting in low-noise tracking. Although long-term changes in body dimensions and skin growth may eventually necessitate reimplantation, we have not observed decline in quality tracking over several weeks of regular use. This straightforward solution enables stable long-term tracking, making it suitable for extended studies of motor learning, adaptation, or other long-term behavioral processes.

### Marker-based versus markerless methods and data quality considerations

The quality and resolution of kinematic data fundamentally determine the scope of behavioral questions that can be addressed. The accuracy and precision of markerless methods inherently depend on video resolution, training datasets, and the consistency of human annotations used during model training. Although extremely high precision with markerless tracking is theoretically achievable, practically replicating the subtle kinematic findings presented here (e.g., differences in MI during stationary periods) would require exceptionally high-resolution video data.

The significant differences observed between CP and vehicle groups involved movements during stationary periods averaging approximately 0.6 mm per 10 frames, and this difference becomes unresolvable when simulating an increase in the recording’s “noise floor” to just 0.65 mm (Fig. 4*a*). Reliably resolving such subtle displacements demands spatial resolution at least half of this magnitude (around 0.3 mm per pixel) with unambiguously identifiable anatomical landmarks. Given our recording arena size (30 × 30 cm), capturing this resolution throughout the arena roughly corresponds to standard high-definition (1080 p) video resolution. However, practical considerations—including camera placement, lens distortion, animal movement across the field, compression artifacts, and inefficient use of the camera’s full field of view—typically necessitate even higher resolution. Even conservatively recorded 1,080 p, 16-bit grayscale videos captured at 300 fps and compressed losslessly typically produce around 20–30 GB of data per minute. In contrast, MBMC datasets remain orders of magnitude smaller—typically tens of MB per minute per marker, even including full 3D reconstruction at 300 fps.

Processing large, high-resolution video files required for subtle-movement analysis also poses practical challenges. Annotating frames for model training or performing inference at full resolutions without downsampling quickly becomes computationally cumbersome. Extending markerless tracking methods into full three-dimensional reconstruction, such as with AniPose (Karashchuk et al., 2021), further amplifies computational complexity and processing demands, significantly increasing both computational burden and data management challenges.

In this study, we instead demonstrate that MBMC yields highly accurate, low-noise trajectories in compact form, immediately ready for analysis after straightforward trajectory labeling. Only minimal data cleaning procedures were applied, such as interpolation across short gaps and occasional single-frame jitter removal, ensuring the captured behavioral features remain free from processing artifacts. Crucially, the inherently low noise floor (quantified here as triangulation residuals) substantially facilitates advanced analytical approaches such as dynamic embedding, which rely on precise characterization of subtle and rapid corrective movements. Such detailed analyses often become impossible or highly error-prone at noise levels that necessitate temporal filtering (Kristianslund et al., 2012).

Although establishing an MBMC system is somewhat more involved than recording with a single camera for markerless tracking, the precision obtained significantly outweighs this initial effort. The accuracy and noise sensitivity of this method enable exploration of subtle behavioral features previously inaccessible with conventional tracking. For example, small-amplitude oscillatory movements of shoulder blade markers during passive immobility potentially reflect breathing, suggesting possible applications in monitoring physiological states such as arousal. Similarly, MBMC robustly resolves subtle, full-body tremors induced by harmaline administration, revealing their spatial and temporal characteristics across the body (Fig. 7). Such tremors, characterized by small amplitudes and high-frequency oscillations, would likely be inaccurately quantified by conventional markerless approaches.

Ultimately, while MBMC is not proposed as a universal replacement for markerless methods, it clearly excels in contexts requiring detailed, precise, and noise-sensitive kinematic analyses of animals moving freely in 3D environments.

In the following, we briefly elaborate on key insights gained from the proof-of-concept experiments presented in this manuscript.

### Context-dependent effects of low-dose CP55,940 on locomotion

A central insight from our experiments is that the behavioral impact of pharmacological interventions depends on the locomotory context. Consistent with previous reports (Patel and Hillard, 2001; Ignatowska-Jankowska et al., 2015a), administration of the cannabinoid receptor agonist (“CP”) slightly reduced overall locomotion in the OF arena (Fig. 3). However, this suppression was not observed when the same mice were tasked with a vertical CLB task on the wheel.

Unlike in OF, CP treatment reduced both the amplitude and speed of hindlimb movements during CLB task (Fig. 6*c,d*). However, this reduction did not alter the ability of the mice to climb, a behavior in which they are naturally skilled, highlighting the multifaceted nature of locomotion. In the more challenging and artificial locomotory task (TRM), CP-treated mice were unable to maintain locomotion at speeds above 20 m/min, suggesting that the same drug-induced reduction in limb vigor had a more pronounced impact under conditions demanding sustained high-speed locomotion.

In contrast, limb kinematics were unaffected in the OF, where locomotion was slower and less demanding (Fig. 6*a,b*) even though CP reduced non-locomotory movements during stationary periods, as indicated by a lower average speed of all markers (quantified as MI; Fig. 4). These findings suggest that the apparent “locomotory suppression” induced by low-dose CP arises from different mechanisms depending on the behavioral context: in the OF, it can reflect motivational changes that reduce levels of exploratory activity, while in CLB or TRM running, it is likely the result of bradykinesia or reduced muscle tone that might only become functionally significant in more demanding tasks.

### Not all swings are the same—insights from peak swing speed timing

Another novel insight emerged when comparing the variation in limb speeds in different locomotory contexts. During running in the TRM task, the ankle speed consistently peaked at the beginning and end of the swings, aligning with the presumed forceful contact of the limb with the moving surface (Fig. 5*c*). In contrast, during OF exploration, ankle speed peaked during the early downswing phase, while it shifted to the upward swing phase in the CLB task. These context-dependent differences in ankle speed timing underscore a key advantage of MBMC: the ability to directly observe and quantify the precise temporal structure of limb movements in animals exploring a relatively broad volume.

Although 3D forelimb speed trajectories have previously been reported and examined in the context of circuit-level disruptions (Becker and Person, 2019; Machado et al., 2020; Calame et al., 2023), the studies typically involved constrained conditions. To our knowledge, we are the first to demonstrate distinct limb timing profiles in the same animals during different, unconstrained locomotor behaviors. In particular, CP administration did not alter the timing of maximum ankle speed during locomotion at self-driven speeds, possibly indicating that the observed reductions in swing vigor may result from effects targeting peripheral circuits or muscles rather than timing mechanisms within central motor circuitry.

In sum, analyzing behavior across multiple, naturalistic locomotory contexts demonstrates that a pharmacological manipulation can produce divergent outcomes and that restricting analysis to a single behavior risks oversimplified conclusions.

### Tracking tremor

Tremor, a rhythmic oscillation of body parts, is a defining symptom of many neurological disorders, including PD and ET (Rahimi et al., 2015; Welton et al., 2021; Angelini et al., 2024). Characterizing the spatiotemporal structure of tremor can provide insight into the underlying mechanisms driving these pathological oscillations.

In animal models, tremor is often quantified using force plates or single-point measurements. While these approaches provide useful metrics, they collapse the complexity of tremor into a single value, obscuring differences and interactions between body parts. We hypothesized that full-body harmaline-induced tremor could be dominated by activity in proximal or larger muscle groups, with oscillatory waves propagating outward to more distal regions. In contrast to this expectation, we found strong in-phase correlation across all tracked markers in all four animals (Fig. 7*g,h*). This is in line with harmaline tremor being mainly expressed through central, bilateral mechanism rather than localized, periferal mechanisms that would propagate the tremor through the neuromotor apparatus (Hopfner and Helmich, 2018; Pan and Kuo, 2018).

Changes in tremor amplitude with respect to behavioral modes can provide valuable insights for characterizing tremor phenotypes. ET typically intensifies during action (e.g., maintaining a posture or performing a movement), whereas Parkinsonian tremor is more prominent at rest and often diminishes during voluntary movement (Deuschl et al., 1998). In our data, harmaline-induced tremor was most prominent during stationary periods, particularly in the one mouse in which it was possible to compare locomotion and immobility (Fig. 7*f*). We found that the tremor subsided during stepping movements, in conflict with the classical features of the ET, albeit possibly reflecting the effect of weight loading and therefore suggesting the involvement of peripheral mechanisms (Hallett, 1998; der Stouwe et al., 2016; Hopfner and Helmich, 2018). Furthermore, we observed bradykinesia-like slowness on the treadmill, possibly related to challenges in the precise timing of limb movements (Fig. 8*c*–*e*).

These findings highlight the bilateral nature of harmaline tremor, consistent with ET, and also reveal distinct behavioral context dependence and possible bradykinesia. Rather than argue for or against the use of harmaline tremor as a model of ET (Louis, 2014), our results underscore the importance of evaluating tremor models in different behavioral models and considering the coordination of tremors throughout the body.

### Concluding remarks

No single approach suits every behavioral study. MBMC, as presented here, excels when the goal is to obtain precise, high-quality kinematics under diverse naturalistic conditions. In contexts where detailed 3D kinematics are unnecessary, when throughput takes priority over accuracy, or when external markers cannot be securely or ethically implanted (e.g., on soft-bodied or wild animals), markerless methods remain appropriate. Similarly, marker implantation may be superfluous if the experimental design inherently restricts animal movements (e.g., head-fixing for two-photon imaging) or focuses primarily on broad movement parameters such as animal location, speed, or proximity to conspecifics.

When detailed and precise 3D kinematic information is required from freely moving animals, MBMC provides distinct advantages by minimizing the need for extensive data post-processing, training, or parameter tuning. This approach yields compact, ready-to-use trajectory data with minimal artifacts, facilitating advanced mathematical analyses. Moreover, the compactness of MBMC data significantly reduces the storage and management demands typically associated with large video files acquired during markerless experiments, while also enabling real-time integration into closed-loop experimental paradigms.

**Figure 9.**
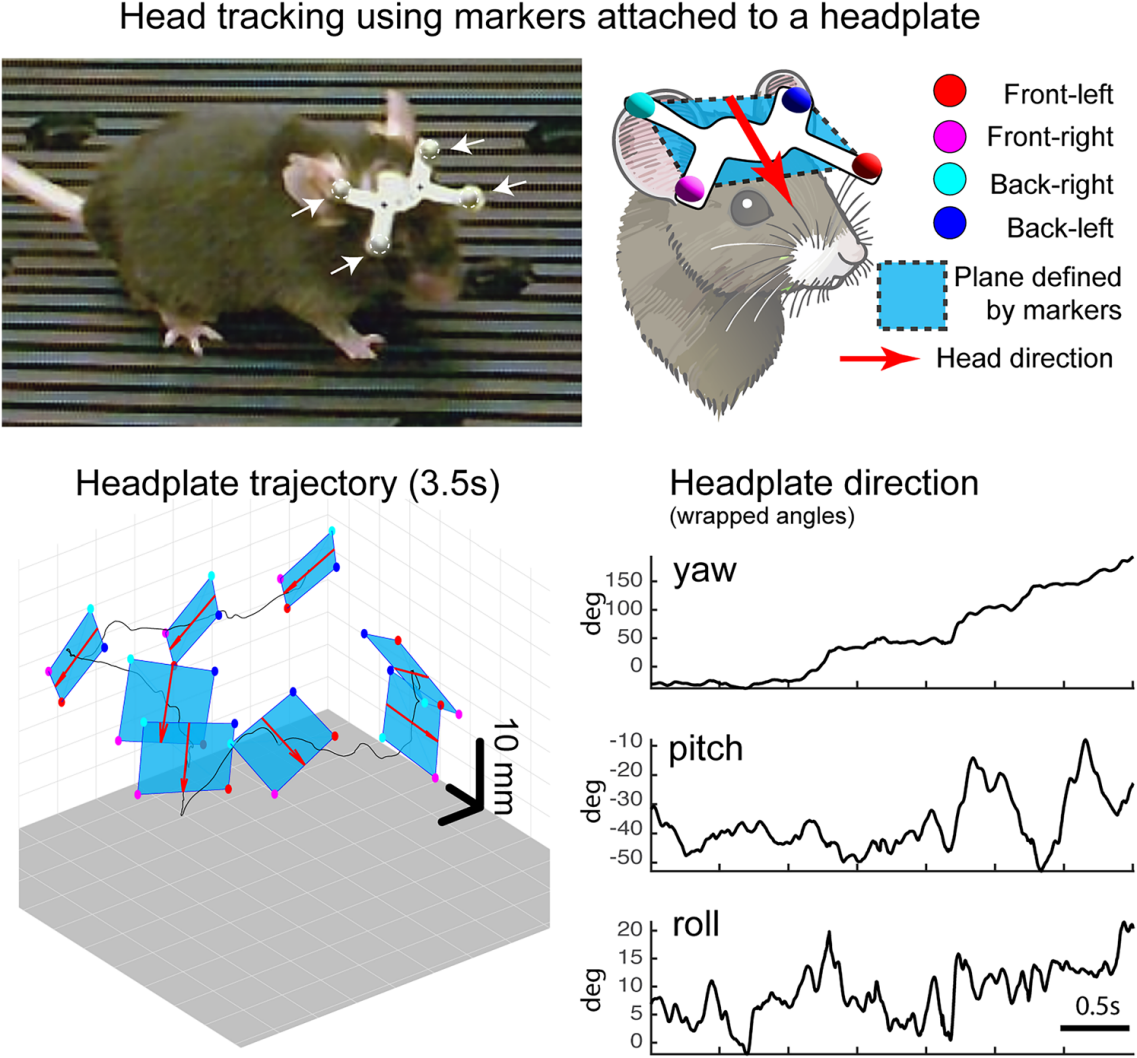
Example of the potential of MBMC in tracking head movements. A conventional headplate (Model 10, Neurotar) is attached to the skull with dental cement. 2.5 mm-diameter retroreflective facial markers (purchased from Qualisys) are attached to the corners of the headplate (top left), thus allowing definition of the 2D plane of head direction (top right). Red arrow depicts “head direction” used in analysis. Bottom left: example trajectories from a 3.5 s long segment of OF exploratory behavior in which mouse turned around. Black line: continuous trajectory of the headplate center; blue rectangles and red arrows depict the head plane and direction, calculated at 0.5 s intervals. Bottom right: head direction representation from the same period, shown in yaw, pitch, and roll angles.

Although MBMC inherently requires physical placement of markers, their positioning is adaptable, allowing straightforward extension beyond the marker configurations demonstrated here. In this study, we utilized markers placed on hind-body and shoulder blade regions, sufficient to address our specific research questions regarding subtle differences in limb kinematics. However, markers can readily be implanted on other body parts such as forelimbs (Movie 13), and precise head orientation tracking can be realized by attaching markers to a lightweight headplate (Headplate Model 10, Neurotar; Fig. 9; combined weight less than 1.1 g)—instead of relying on head-mounted inertial measurement units (Wilson et al., 2018; González-Rueda et al., 2024). Importantly, MBMC implementation is not restricted to high-end motion capture systems such as Qualisys, Vicon (https://www.vicon.com/), or OptiTrack (https://optitrack.com/); flexible and low-cost hardware alternatives are also available (Chatzitofis et al., 2021).

**Movie 13.** Video and motion capture reconstructions of mice locomoting with forelimb or headplate trackers. [View online]

The utility of MBMC extends naturally to longitudinal tracking across the lifespan of individual animals, providing unique opportunities for within-subject analyses over extended periods. Similarly, the approach can be readily adapted for multi-animal tracking and offers potential in studying disorders such as dystonia, epilepsy, or autistic phenotypes, where fine-grained motion analysis could uncover more subtle patterns than those detectable with existing methods (Cook, 2016; Velíšková and Velíšek, 2017; Streng et al., 2021; Klibaite et al., 2022; Snell et al., 2022; Gray et al., 2023; Gschwind et al., 2023; van der Heijden et al., 2024; Washburn et al., 2024).

Despite these clear advantages, MBMC involves certain practical considerations. Setting up multi-camera systems requires initial technical investment and expertise, and marker implantation—although minimally invasive and straightforward—necessitates brief anesthesia and appropriate surgical precautions. Additionally, successful MBMC recording outside conventional enclosures requires careful experimental planning with the animals’ well-being in mind. Experimenters must remain mindful of animal comfort and maintain consistent procedures to ensure stable and low stress behavior. Although this initial effort may exceed that deemed sufficient in more conventional setups, it significantly reduces stress-induced behaviors, enhancing the validity and quality of the obtained kinematic data.

We hope that our practical implementation of MBMC in mice, supported by the detailed animal-training guidelines provided (Methods, 3.3), will motivate new analytical approaches to exploring detailed, full-body dynamics of mouse behavior in species-appropriate contexts.

### Ethical Approval Declarations

The animal study protocol was conducted in accordance with procedures approved by the Okinawa Institute of Science and Technology (OIST) Institutional Animal Care and Use Committee (IACUC) (Protocol IDs: 2017-188, 2020-305) in accordance with the National Institutes of Health Guide for the Care and Use of Laboratory Animals (National Research Council, 2011). Every effort was made to minimize suffering and discomfort.

## Data Availability

Trajectory data in non-processed form will be made available at ZENODO http://doi.org/10.5281/zenodo.15493339.

## Code Availability

All code for result generation will be made available at https://github.com/nRIM-OIST/Marker-based-Mouse-MoCap-eNeuro-2025.

## Synthesis

Reviewing Editor: Arvind Kumar, KTH Royal Institute of Technology Decisions are customarily a result of the Reviewing Editor and the peer reviewers coming together and discussing their recommendations until a consensus is reached. When revisions are invited, a fact-based synthesis statement explaining their decision and outlining what is needed to prepare a revision will be listed below. The following reviewer(s) agreed to reveal their identity: Luciano Censoni.

Synthesis

We apologize for the delay in the processing of this manuscript. It was surprisingly difficult to find reviewers and when we did secure two reviewers, one of them eventually did not respond. So we have to now make the decision based on one report.

The reviewer is generally positive about the work but he/she has raised some concerns about the claimed efficacy of the method i.e. the limitations of the proposed methods should be discussed. In addition, some methodological information and appropriate controls are also missing.

The comments from the reviewer are appended below to help you revise the manuscript.

Reviewer comments

The authors describe a novel approach to marker-based motion capture in mice, through the implantation of (standard, human skin decoration-type) piercings which can support modified retroreflective ends, which can subsequently be tracked with standard commercial, multi-camera solutions during behavioral experiments.

The authors correctly point out that markerless tracking, in particular using free, deep-learning based approaches such as the DeepLabCut package, has become the de facto standard for kinematic tracking in mice, but that these approaches often require extensive post-processing and can nevertheless suffer from jitter that makes the analysis of fine-scale kinematics impossible.

In this context, the authors offer marker-based motion capture, enabled by their resourceful marker construction and implantation protocol, together with the use of existing commercial, multi-camera tracking systems, as an alternative that can be superior to markerless tracking, in particular for detailed investigations of fine-scale kinematics. However, in the absence of head-to-head comparisons with a markerless tracking approach, the manuscript falls just short of establishing that the level of kinematic detail revealed by this approach was in fact previously inaccessible.

I believe this manuscript is of interest for the eNeuro readership, as it describes a potentially important methodological advance (if somewhat restricted in terms of setup requirements), but that it would be significantly improved by the addition of direct comparisons between the results obtained with this novel approach and the previous state-of-the-art.

Below, I offer a list of specific feedback points that I would like to see addressed:

Major points

1) It is unclear if the paragraph between lines 115-125 is sufficiently supported. The outputting of "compact" 2D coordinates from high-resolution images is a standard feature of markerless systems such as, e.g., DeepLabCut.

Moreover, the authors claim that the dedicated infrared illumination eliminates noise related to changing light conditions - a real issue that forces setups that rely on markerless tracking to keep light conditions carefully controlled -, but the authors later point out (see line 1654) that the required absence of a conventional enclosure mandates carefully controlled external conditions, including lighting, in order not to negatively influence the animal behavior, potentially negating the advantage claimed here.

I suggest the authors add further references to support the claims made in this paragraph, while addressing the caveats mentioned above, or perhaps suppress this paragraph, which could be done without impacting the points raised in the introduction.

2) The required absence of conventional enclosures (line 159) could be considered an important disadvantage of this approach, but this is not brought up sufficiently clearly or discussed outside of the methods section (see line 1475). I suggest that the authors include a brief mention of this requirement in the introduction and a short discussion of its consequences in the discussion section.

3) The weight of the implants is not mentioned when they're described in the results section (paragraph starting at line 220), but it is an important factor in determining whether they interfere with the animal's behavior or the kinematics of particular movements. In the methods section the authors clarify that the implants weigh in total no more than 10% of the animal's body weight (line 1462), but it is unclear if that figure refers to the weight of the implants with the standard ends or with the custom-made retroreflective ends which are used during data acquisition and which the authors mention are larger and heavier (line 2401).

I suggest that the authors mention the weight of the implants when they're first described in the results section, and clarify if the "10%" figure also applies to the actual ends used during experimental conditions.

Furthermore, the authors may want to discuss if it would be possible to apply the implants to the animal head and forelimbs and head as well, which could make them significantly more useful in the study of naturalistic behavior such as grooming and/or particular disease models such as Levodopa-induced dyskinesia.

4) The description of the "residual triangulation" value (line 369, again in line 1719) is unclear for a reader that is not familiar with how the commercial multi-camera setup works. The precision calculated here underpins the assertion that the reported approach can access ground truth at submilimiter accuracy (line 1055). Therefore, I suggest that the authors clarify this calculation.

5) This manuscript would be substantially improved by the addition of head-to-head comparisons with a markerless system. For instance, our labs's experience suggests that the results described in sections 3.3 and 3.4 could be relatively simple to reproduce using e.g. DeepLabCut and a single, appropriately-angled camera, and are, therefore, not necessarily an improvement upon the state-of-the-art. On the other hand, the results showcased in 3.5-3.9 might indeed be harder to reproduce using a markerless setup.

Adding such control experiments would certainly strengthen the overall argument. Otherwise, I suggest that the authors weaken some of the assertions regarding previous inaccessibility of behavioral features, e.g. the paragraph starting at line 1175.

Minor points/typos

1) In line 316 as well as all subsequent mentions of durations given in frames, please add the corresponding durations in seconds; camera framerates are currently not described outside of the methods section.

2) Typos:

Line 082 - the authors presumably meant "markerless" instead of "marker-based"

Line 199 - typo: "realiable"

Line 697 - typo: "corridoors"

Line 754 - "ask" presumably should have been "task"

Line 846 - a ")" is missing.

Line 2456 - "ame" should be "same"

## Author Response

# Reviewer \#1 **Reviewer comment:** The authors describe a novel approach to marker-based motion capture in mice, through the implantation of (standard, human skin decoration-type) piercings which can support modified retroreflective ends, which can subsequently be tracked with standard commercial, multi-camera solutions during behavioral experiments.

The authors correctly point out that markerless tracking, in particular using free, deep-learning based approaches such as the DeepLabCut package, has become the de facto standard for kinematic tracking in mice, but that these approaches often require extensive post-processing and can nevertheless suffer from jitter that makes the analysis of fine-scale kinematics impossible.

In this context, the authors offer marker-based motion capture, enabled by their resourceful marker construction and implantation protocol, together with the use of existing commercial, multi-camera tracking systems, as an alternative that can be superior to markerless tracking, in particular for detailed investigations of fine-scale kinematics. However, in the absence of head-to-head comparisons with a markerless tracking approach, the manuscript falls just short of establishing that the level of kinematic detail revealed by this approach was in fact previously inaccessible.

I believe this manuscript is of interest for the eNeuro readership, as it describes a potentially important methodological advance (if somewhat restricted in terms of setup requirements), but that it would be significantly improved by the addition of direct comparisons between the results obtained with this novel approach and the previous state-of-the-art.

Below, I offer a list of specific feedback points that I would like to see addressed: **Author response:** We are grateful for the reviewer's encouraging words and thoughtful attention to the issues in the manuscript, that we have worked on to the best of our ability. Please find our point-by-point responses below. ## Major comments **Reviewer comment:** 1. It is unclear if the paragraph between lines 115-125 is sufficiently supported. The outputting of "compact" 2D coordinates from high-resolution images is a standard feature of markerless systems such as, e.g., DeepLabCut. **Author response:** We apologize for not clearly describing our intent. An important feature of marker-based motion capture (MBMC) with dedicated motion capture cameras is that they , entirely removing the necessity of storing and processing high-resolution video recordings. For comparison, high-resolution, high-framerate (e.g., 100 fps) videos required to capture movements of freely behaving animals typically produce file sizes of approximately 30 GB per minute, while the raw trajectory data from motion capture systems typically result in filesizes around tens of MB per minute (e.g. 32bit coordinate + residual data, at 300 fps). Notably, such compact trajectory coordinates can be streamed nearly-real-time into e.g. closed-loop control applications, a feat that is relatively challenging with markerless methods (Kane et al, 2020, PMID 33289631). Furthermore, although file size is not directly related to scientific rigor, data storage, management (including backups), and reuse of large-scale research datasets are practical challenges that may necessitate restrictions on data size in the future (see e.g. ).

Importantly, when using an MBMC system like ours, where raw videos are not stored, it is crucial to always record a synchronized, high-quality conventional video alongside the motion capture data.

We added the word "on-camera" to the mention of data compression, mention the necessity of complementing the motion capture data with one well-placd camera (Methods, section 5.6) as well as further elaboration of the benefit of this aspect in the discussion (last paragraph). Furthermore, we added the missing mention of the conventional video camera in results (section 3.1). **Reviewer comment:** Moreover, the authors claim that the dedicated infrared illumination eliminates noise related to changing light conditions - a real issue that forces setups that rely on markerless tracking to keep light conditions carefully controlled -, but the authors later point out (see line 1654) that the required absence of a conventional enclosure mandates carefully controlled external conditions, including lighting, in order not to negatively influence the animal behavior, potentially negating the advantage claimed here.

I suggest the authors add further references to support the claims made in this paragraph, while addressing the caveats mentioned above, or perhaps suppress this paragraph, which could be done without impacting the points raised in the introduction. **Author response:** We appreciate the reviewer pointing out the potential confusion regarding lighting controls. Indeed, environmental conditions such as lighting affect both markerless and marker-based approaches, but the scale and sensitivity differ significantly. Markerless tracking typically requires meticulous control of subtle lighting parameters (e.g., minor shifts in illumination intensity, LED positioning, or shadows), as even slight variations can introduce substantial tracking artifacts or jitter, significantly reducing data quality. Although DeepLabCut and similar markerless methods perform well under variable conditions when these are comprehensively represented in their training data, unplanned or subtle environmental changesâ€"such as progressive dimming of a light source due to surface soilingâ€"can still negatively impact tracking quality.

In contrast, for marker-based motion capture (MBMC), dedicated infrared illumination ensures stable tracking independently of these minor lighting fluctuations. The environmental controls we emphasize for MBMC specifically address avoiding sudden or drastic changes (such as abrupt turning lighting off or on, loud noises or sudden movements by personnel), which could otherwise startle or stress the animals. Thus, the experimental precautions we takeâ€"stable general lighting, minimal noise, and restricted personnel movementâ€"primarily aim to maintain animal comfort and ensure naturalistic behavior, rather than being driven by the tracking technology itself. Importantly, the level of environmental control needed depends largely on animal habituation; in our experience, thoroughly habituated animals tolerate moderate environmental variations without significant behavioral disruption. We have explicitly clarified this distinction in the Methods section of the revised manuscript and added a brief mention regarding animal habituation and handling in the Introduction (around line 162). **Reviewer comment:** 2) The required absence of conventional enclosures (line 159) could be considered an important disadvantage of this approach, but this is not brought up sufficiently clearly or discussed outside of the methods section (see line 1475). I suggest that the authors include a brief mention of this requirement in the introduction and a short discussion of its consequences in the discussion section. **Author response:** We thank the reviewer for highlighting the need for more detailed discussion of important point. To explicitly address this, we have included clear mentions of the absence of conventional enclosures and the necessity of making use of appropriate habituation procedures, in both the Introduction and the Discussion sections. Specifically, we added a sentence in the Introduction (around line 165) clarifying that MBMC recording requires careful animal habituation, consistent handling routines, and familiarization of experimenters with working without enclosures. Moreover, we significantly restructured our concluding remarks (section 4.6) to include a dedicated paragraph explicitly discussing practical considerations and caveats associated with performing MBMC without conventional enclosures. In this paragraph, we emphasize that successful recordings rely on careful experimental planning with animal well-being in mind, consistent procedures, and mindful animal handling practices that help minimize stress-induced behaviors, thereby improving data validity. Notably, we provide detailed guidelines in the methods (Section 5.3) that describe the handling and habituation protocols. **Reviewer comment:** 3) The weight of the implants is not mentioned when they're described in the results section (paragraph starting at line 220), but it is an important factor in determining whether they interfere with the animal's behavior or the kinematics of particular movements. In the methods section the authors clarify that the implants weigh in total no more than 10\% of the animal's body weight (line 1462), but it is unclear if that figure refers to the weight of the implants with the standard ends or with the custom-made retroreflective ends which are used during data acquisition and which the authors mention are larger and heavier (line 2401).

I suggest that the authors mention the weight of the implants when they're first described in the results section, and clarify if the "10\%" figure also applies to the actual ends used during experimental conditions. **Author response:** We apologise for the oversight. The reviewer is correct in that the weight of the retroreflective marker is significantly larger than that of the bare steel base (averages 95 vs 240 mg). We have revised section 3.1 by adding details of the marker weights in the text (results lines 220-221, 238- ). Also we added words specifying the 10 \% of body weight (actually, 5-7 \%) relates to "uncoated" markers (in Methods, line 1480). At the time of the recordings (at least 2 weeks after implantation), mice weigh more than 25g.

Finally, we note that it is fundamentally impossible for us to unequivocally prove that marker attachment does not lead to changes in fine kinematics, there is no method for comparing the kinematics of freely moving animals obtained with marker-based and markerless methods. The best we can do is examine whether there are visible visible behavioral changes. In this vein, we have observed that switching the small spheres to heavier ones does not affect the capacity of mice to run at high speeds on a motorized treadmill. In revision we first included supplementary data demonstrating that the coated steel markers do not impair locomotion in treadmill experiments (N = 40 mice, tested with light and heavy markers consequently, p=0.08). But after noticing that eNeuro does not permit supplementary materials, we have removed this (as well as supplementary figures 1-3 that in our view do not belong in main figures). Since this analysis was performed as part of another ongoing study involving particular treadmill behavior (not otherwise included in this manuscript), we felt it was more appropriate to remove the figure altogether rather than try to incorporate it. If the reviewer feels showing this data is critical, we are happy to include it in a main figure panel, though we opted not to do so here in order to keep the main narrative focused. **Reviewer comment:** Furthermore, the authors may want to discuss if it would be possible to apply the implants to the animal head and forelimbs and head as well, which could make them significantly more useful in the study of naturalistic behavior such as grooming and/or particular disease models such as Levodopa-induced dyskinesia. **Author response:** In fact we are in other projects already using tracking of forelimbs and the head. Obviously, for the comfort of the animal it is best to avoid attaching more markers than is necessary for answering the question at hand, and in the context of locomotion we have found that shoulderblades provide sufficient amount of insight into the frontlimb movements. We have added a new figure 9 describing head-tracking, and a new Movie 13 showing examples of forelimb tracking; also, we have added discussion to section 4.3 describing these possibilities.

It is our position that while marker-based tracking of individual digits could be attainable under restricted conditions, investigation of manual dexterity in mice requiring single-digit tracking is most likely best conducted with markerless methodologies. In contrast, marker-based approach is appropriate when fine kinematics are investigated in a context where movement restriction would disrupt the behavior. **Reviewer comment:** 4) The description of the "residual triangulation" value (line 369, again in line 1719) is unclear for a reader that is not familiar with how the commercial multi-camera setup works. The precision calculated here underpins the assertion that the reported approach can access ground truth at submilimiter accuracy (line 1055). Therefore, I suggest that the authors clarify this calculation. **Author response:** We thank the reviewer for pointing out this lack of clarity. We have now expanded and clarified the description of the "residual triangulation" metric in both the Results (line 389-) and Methods (1682-) sections. Specifically, we now explicitly state that residual triangulation represents the discrepancy between reconstructed 3D marker positions and their underlying 2D projections from each camera view. Additionally, we provide a standard reference on multicamera geometry and triangulation (Hartley and Zisserman, 2003). We hope this makes the concept clearer and helps readers understand the technical foundations behind the claimed tracking accuracy. **Reviewer comment:** 5) This manuscript would be substantially improved by the addition of head-to-head comparisons with a markerless system. For instance, our labs's experience suggests that the results described in sections 3.3 and 3.4 could be relatively simple to reproduce using e.g. DeepLabCut and a single, appropriately-angled camera, and are, therefore, not necessarily an improvement upon the state-of-the-art. On the other hand, the results showcased in 3.5-3.9 might indeed be harder to reproduce using a markerless setup.

Adding such control experiments would certainly strengthen the overall argument. Otherwise, I suggest that the authors weaken some of the assertions regarding previous inaccessibility of behavioral features, e.g. the paragraph starting at line 1175. **Author response:** We thank the reviewer for this important and insightful comment. We fully agree that markerless methods, such as DeepLabCut, can indeed capture many basic kinematic measures, especially general parameters like position, speed, or broad locomotory activity. In fact, we do not propose MBMC as a replacement for markerless methods but rather suggest it as an enabler of entirely new experimental directions by allowing more direct observation of fine movements during unrestricted behaviors.

To more clearly illustrate this distinction, we have added a new panels to figure 4, explicitly demonstrating the minimal scale of movements (approximately 0.6 mm per 10 frames) underlying the statistically significant differences observed in the "Motion Index" during stationary periods (Section 3.4). We also now thoroughly discuss practical considerations associated with markerless approachesâ€"particularly the trade-offs between resolution, computational complexity, and required data handling capabilitiesâ€"in the Discussion section ("Marker-based versus markerless methods and data quality considerations").

As the reviewer correctly points out, it remains theoretically possible that markerless methods could replicate these results given sufficiently high-quality video data, improved camera equipment, and extensive model training. Thus, definitively "proving" that markerless methods cannot match MBMC in these contexts is inherently challenging, as one could always argue for better resolution, improved model training, or more computational resources. Our intention is not to assert MBMC as universally superior, but rather to clearly illustrate contexts where its distinct advantagesâ€"particularly its inherently low noise floor and high spatiotemporal resolutionâ€"become practically and analytically valuable. For instance, subtle, high-frequency tremors as shown in Figure 7 exemplify scenarios in which MBMC excels by providing detailed spatiotemporal data that conventional markerless setups would likely struggle to capture reliably without substantial technological or computational overhead.

At present, we do not have access to camera systems that would enable direct, high-resolution comparative recordings under freely-moving conditions. Consequently, we are unable to provide direct empirical comparisons between marker-based and markerless methods. However, to explicitly address the reviewer's suggestion, we have added detailed elaborations to Sections 3.3 and 3.4, clearly acknowledging that general locomotory parameters (as illustrated in Figure 3) could indeed be captured by simpler or markerless methods. Indeed, a key point we inadvertently omitted previously â€" and now explicitly emphasize, also in response to Major Comment 3 â€" is the rationale for analyzing these general movement parameters. As the reviewer insightfully notes, it is essential to examine whether wearing markers leads to overt aberrations in animal behavior. The locomotory effects presented in Figure 3 align closely with earlier findings obtained using traditional methods, thereby providing at least preliminary confidence that marker attachment does not significantly disrupt normal behaviors.

We have further clarified our statements regarding the universal accessibility of certain behavioral features, explicitly positioning MBMC as a complementary method highly advantageous in specific contexts requiring precise, detailed, and noise-sensitive kinematic analyses of freely moving animals (in Abstract, Introduction, Results and Discussion). We hope these adjustments thoroughly address the reviewerâ€{trade mark, serif}s thoughtful and constructive feedback. ## Minor comments **Reviewer comment:** 1) In line 316 as well as all subsequent mentions of durations given in frames, please add the corresponding durations in seconds; camera framerates are currently not described outside of the methods section. **Author response:** Apologies for omitting this important information from the main text. We have added the durations provided in frames also in milliseconds. However, since we do not have the measurements of sub-millisecond temporal accuracy of camera shutters, we provide the values rounded to the nearest millisecond. **Reviewer comment:** 2) Typos:

Line 082 - the authors presumably meant "markerless" instead of "marker-based" Line 199 - typo: "realiable" Line 697 - typo: "corridoors" Line 754 - "ask" presumably should have been "task" Line 846 - a ")" is missing.

Line 2456 - "ame" should be "same" **Author response:** We thank the reviewer for careful reading. We have corrected all these typos. We also have found others that are now corrected.
